# Supplementary material for: Differential CaKAN3-CaHSF8 associations underlie distinct immune and heat responses under high temperature and high humidity conditions
Source: Nat Commun. 2023 Jul 25;14:4477. doi: 10.1038/s41467-023-40251-8 (PMC10368638; doi:10.1038/s41467-023-40251-8)
Supplement: Supplementary file 1 — Supplementary Information [file 41467_2023_40251_MOESM1_ESM.pdf]

Tables

Supplementary Data Table 1. Primers used in this study.

	Gene	F primer	R primer
**Primers used for *CaKAN3* and *CaHSF8* study**	***CaKAN3***	GGGGACAAGTTTGTACAAAAAAGCAGGCTTCATGATGGAATATTTCTCAAT	GGGGACCACTTTGTACAAGAAAGCTGGGTCTTAACAATAGAAATATGAAG
	***CaKAN3*-GFP**	GGGGACAAGTTTGTACAAAAAAGCAGGCTTCATGATGGAATATTTCTCAAT	GGGGACCACTTTGTACAAGAAAGCTGGGTCACAATAGAAATATGAAG
	***CaKAN3*-VIGS**	GGGGACAAGTTTGTACAAAAAAGCAGGCTTC AAGGGAGAACAATATTTGGC	GGGGACCACTTTGTACAAGAAAGCTGGGTCTTTCTCACTAACCAAAGGAC
	***CaHSF8*-VIGS**	GGGGACAAGTTTGTACAAAAAAGCAGGCTTC TGAGTAGCTCAAATGCGCCA	GGGGACCACTTTGTACAAGAAAGCTGGGTCTCTGTCCATGAGCAGGCTTG
	***CaKAN4*-GFP**	GGGGACAAGTTTGTACAAAAAAGCAGGCTTCATGATGAAAAAAATATTCAT	GGGGACCACTTTGTACAAGAAAGCTGGGTCGGGAAAAGTTTCTTCAAAAC
	CaHSF8	GGGGACAAGTTTGTACAAAAAAGCAGGCTTCATGGGTTCTGCTTCAATGGA	GGGGACCACTTTGTACAAGAAAGCTGGGTCTCATACTTTTTTACTGTTTG
	***CaHSF8*-GFP**	GGGGACAAGTTTGTACAAAAAAGCAGGCTTCATGGGTTCTGCTTCAATGGA	GGGGACCACTTTGTACAAGAAAGCTGGGTCTACTTTTTTACTGTTTG
	***CaHSF8*-D1**	GGGGACAAGTTTGTACAAAAAAGCAGGCTTCATGGGTTCTGCTTCAATGGA	GGGGACCACTTTGTACAAGAAAGCTGGGTCTCAGCGCCTACTAATATTTCTAA
	***CaHSF8*-D2**	GGGGACAAGTTTGTACAAAAAAGCAGGCTTCATGGGTTCTGCTTCAATGGA	GGGGACCACTTTGTACAAGAAAGCTGGGTCTCACAGTAAACTTTCAGGACTGT
	***CaHSF8*-D3**	GGGGACAAGTTTGTACAAAAAAGCAGGCTTCATGGGTTCTGCTTCAATGGA	GGGGACCACTTTGTACAAGAAAGCTGGGTCGTTTTGAAGAAACTTTTCCC
	***CaKAN3*^{myb}**	GGGGACAAGTTTGTACAAAAAAGCAGGCTTCATGATGGAATATTTCTCAAT	GGGGACCACTTTGTACAAGAAAGCTGGGTCTTATCTGTACATCTGGAGATGGC
	***CaHSFA1*-GFP**	GGGGACAAGTTTGTACAAAAAAGCAGGCTTCATGCCTTTACACTTCCCTCT	GGGGACCACTTTGTACAAGAAAGCTGGGTCGTTTTGAAGAAACTTTTCCC
**Primers used for pepper q-PCR analysis**	***CaKAN3*-qPCR**	AATCATCAGTTCCTCGTCTT	GTCAATCCCTTTACTTCCATC
	***CaHSF8*-qPCR**	TTGAAGAAGAGGTTGAGAGG	TCGCTGCTCCATAATATGAA
	***CaR1B23*-qPCR**	TGGCCTGGACACCACAATTT	TTGTTTCCGGAAGACCCTCG
	***CaR1B11*-qPCR**	GTACTACCCCGTTGAGCCAC	AGGTGTAGCTCTTGTTCCAACT
	***CaR1A*-qPCR**	AGCTACGACATGTGGACACC	TCTGCTCTGCTGCTGAACAA

	***CaR1B12*-qPCR**	CCTAGACGTGACTTCCGAGC	TCTTCCCAACAATCGCAGCT
	***CaR1A6*-qPCR**	TGTGCATGGGTCACTGTCTC	TCCCAAGCCTCAGTACTCCA
	***CaR1B16*-qPCR**	ATGACACGTCGAGCCCAAAT	ACTACCACCAGCAACCATGG
	***CaHSP17.4B*-qPCR**	TCAGCTTCATCAGATGCACCT	TCGTGGCAAGCAATTCACTT
	***CaHSP70*-qPCR**	GTGCAACTTCCGGTGGAATG	GGACAGTAGGGGTGAGGGAA
	***CaHSP18.2*-qPCR**	TGTGCAGTGACCCTTCAGAA	CGACGATATTCCCCACAAAGC
	***CaHSP70-15*-qPCR**	TGCTGAGCCAATGGAGATGG	TGTTGACTGCTGCTGTAGGT
	***CaPRP1*-qPCR**[1]	ACCAAACTCGGATATTCCAACT	GAGGAATCCTCGGAACCAAGT
	***CaMgst3*-qPCR**[1]	TCCAGCTTTTCGCACTCTCT	CGAGATCTCGCCACCCAATT
	***CaACTIN*-qPCR**[2]	AGGGATGGGTCAAAAGGATGC	GAGACAACACCGCCTGAATAGC
	***NbR1B23*-qPCR**	GATGATGCTTGCTCATCGCT	ACGGCAGGTGACAGCTAAAA
	***NbR1B11*-qPCR**	CCCTTCATGTTGGGGGACTC	TATTGTGTGCCAGGCCACAT
	***NbR1B12*-qPCR**	GAACTCCTACTCCCGCGAAG	CCGGCCATTCCCATAATCGA
	***NbR1A6*-qPCR**	AACGACGGGCTAATGGAAGG	GAAAGCATAGCTCGCCGTTG
	***NbR1B16*-qPCR**	ATGCCCGAGACGCTAACAAA	CCTTGAGGGTCGGAAACTCC
	***NbEF-1a*-qPCR**[2]	TGCTGCTGTAACAAGATGGATGC	GAGATGGGGACAAAGGGGATT
**Primers used for ChIP-PCR**	**pCaR1B23-P1**	AGTCCGGTGCACTAAAGCTC	CCACAAGGGAGAAGGAAAATCC
	**pCaR1B23-P2**	TCCCTGATATCCCTGCATGAC	CGGATTGTCACGGATGATGC
	**pCaR1B23-P3**	CCTTTGCTCTCTCCTTGCAGA	TGCGAAGGTAAGAGCTTGGA
	**pCaR1B23-HSE**	AGAAGGCATCATCCGTGACA	AAGTGGTAGGTGCGTGATGA
	**pCaR1B11-P1**	CCGAACAGTCACTCCTTTTCC	CCATTCACGTGGGGGATTGT
	**pCaR1B11-P2**	GGCTATTGTAACACCCCGCA	TGCCCAACTAGATTCACACACT
	**pCaR1B11-P3**	TGATGAACTTGGGGGCTTACT	TGTTGGAGCAAAATGGGCAT
	**pCaR1B11-HSE**	CCGTCATTTCTAGAAGTAGATGTTCC	TGATCTCGAATCATGCCCAACT
	**pCaR1A-P1**	AGGTTGAACTGAATATTCTTTGTCGA	TCACTGCTATGGAATCTTTCTCA
	**pCaR1A-P2**	ATGAAATTGGGCTACACCGT	ACCCTCTAATGATTGTGCGAA
	**pCaR1A-P3**	GGGTAATGCGCTCCCTAACA	CAAGGATTGTGGGGGAGTAGT
	**pCaR1A-HSE**	CTCCCCCACAATCCTTGTTGA	ACCTTAATATGGCAGTGAGGACT

	**pCaR1B12-P1**	AGTTGGATATATAGTTTCCATCAGCA	TGGTGTCAAGTTTAGGGTCGT
	**pCaR1B12-P2**	ACCTTTGATTTTGCAACTTCATGC	ACCTCTCTCCAAGTCAGCCT
	**pCaR1B12-P3**	TGTGCACTGTCAAAGTTTAAGGT	TGTGAACTCTTCAAACCCCTT
	**pCaR1B12-P4**	AAAGGGGTTTGAAGAGTTCACA	TTAGTTTTGTGCTTTGCAACGA
	**pCaR1B12-P5**	TCTTCCCAACAATCGCAGCT	ACGTGACTTCCGAGCAGTAG
	**pCaR1B12-HSE**	AGTTGGATATATAGTTTCCATCAGCA	TGGTGTCAAGTTTAGGGTCGT
	**pCaR1A6-P1**	TGTCATCTTTCATTTAGACTTTGTTGA	AGTTCATGGTCAAGTCATCCGT
	**pCaR1A6-P2**	TGTGCTTTGTTCCGGGTAATG	ACAAGCAATGCAAGACAGTGT
	**pCaR1A6-P3**	AGCAGGTTCTCGAGTATTCCA	CCTTCCTGTTGATCTAGCTGAGG
	**pCaR1A6-HSE**	GGGGGAAATGAAATTCCTAACCT	CATTACCCGGAACAAAGCACA
	**pCaR1B16-P1**	TCTCAATTCTCACCACCACCA	ACTGCACCTTAGGGGATCTTG
	**pCaR1B16-P2**	TGAAAACAATAACAAGATCCGACCT	AGCTGAGTTCTGCTTTGGACA
	**pCaR1B16-P3**	ACAGCAATTACACTCAACTATCCA	CCTCATTGGTTGCTTGTGTTGT
	**pCaR1B16-P4**	CACAAGCAACCAATGAGGAAA	TCGGTTTTCGTTTGAAAGCACA
	**pCaR1B16-P5**	TGTGCTTTCAAACGAAAACCGA	TGGCTGAAATTCTCATGCTTTCC
	**pCaR1B16-HSE**	TGAAAACAATAACAAGATCCGACCT	AGCTGAGTTCTGCTTTGGACA
	**pCaMgst3-P1**	AAGCTTCCGTCGAAGTTTGC	TCCGTTGACATTGTCCCCTT
	**pCaMgst3-P2**	TCGTTCTTGATAGCAGAACCA	GCGAAACGAATAAATGTTTGTCGA
	**pCaMgst3-P3**	TCCCGTTCTTCATTAAGTTAGTCT	ACGAGTTAAGGACCCGTTTGG
	**pCaMgst3-P4**	TCCAGCTTTTCGCACTCTCT	CGAGATCTCGCCACCCAATT
	**pCaPRP1-P5**	TTGTCGTGTTTGATCCTATC	ACTACCGTAGTGGGAAATTTA
	**pCaPRP1-P6**	AGTCGAAAATTATTTTCCGACAAGC	GACGGCCAATGTAGTCGGAA
	**pCaPRP1-P7**	CCCTTTTAACGACGTGGCAC	TGAACAAATATGACCTCCGATTTTGT
	**pCaHSP17.4B^{AACAA}**	CACAATTCGTGCCTACTTTAAGT	AGTTCATCCCACTTACCGGG
	**pCaHSP17.4B-HSE**	TGCTTCATGTCCCAATGCGA	CGGCAAAAAGGGTTGAGCAA
	**pCaHSP70^{AACAA}**	TCGAAATAACATTATGTGAAGGGTT	TCTTCACATTTTTAATTCGCAGTGT
	**pCaHSP70-HSE**	TCTTGCAGTCTGCTTGGTCA	TGCATGTGATGTGTACTGAGA
	**pCaHSP18.2^{AACAA}**	ACAAAGCCAAGACCCACAAC	AGTGCATGCAGATCAGCTGT
	**pCaHSP18.2-HSE**	GTTTCACCGGGATTCGCTTT	GTTGTGGGTCTTGGCTTTGT

	**pCaHSP70-15^{AACAA}**	GCTATAAGGAGCCAAACACAGC	ACATTATCCTGTTTACCAGAGGGG
	**pCaHSP70-15-HSE**	TTTTCGGTCTGGCAATTCGC	CGCTATCCTTTTTCTTTTTCTGTGC
	**pNbR1B23-P1**	ACGATTTTGCTTTTAAAATTAAGCTGT	ATTGCAGCAACCCAAAGCTC
	**pNbR1B23-P2**	AGGAATCTCGTGCCACATGC	CGAAGGAGAGATGATCTAGTTACCC
	**pNbR1B23-P3**	TGATGCTGCTCTTTGTCCTT	AGTTATCAGCTATCATTAACCACTCA
	**pNbR1B23-P4**	CGTCTGCAACAACTTGGCAA	TGAGGCTCTGCAAGTAGAGA
	**pNbR1B23-PH1**	CAGTGGCTCTCCTTCTTCCC	TGGTTAGAAGACATTCTTTTCTGAGA
	**pNbR1B11-P1**	TTCCGAATTGCTCCGGCTAA	TATCCTCCCCAGACCTCACG
	**pNbR1B11-P2**	TGTTGTTTGACCATCGGCTT	ACCTCAACCATAACTCACGAGG
	**pNbR1B11-P3**	TGACAGAATGAGTTCCAAACCTG	TCATTTCGGATTATGGAGAAGTCA
	**pNbR1B11-P4**	GTCTGTTTCCTTTCACTTGACGG	CCAGGGGTCTAGGAGGCTAA
	**pNbR1B11-PH2**	TCAGTAATTTCGGTTAAGTTGAGAAGT	ATTTTGATATTCGCGCGGGC
	**pNbR1B12-P1**	TGGCACACAATCTCACTTTCTG	ATCCGGGTATAGGTCAGGGG
	**pNbR1B12-P2**	TGCGGGAATCTTATTACCCCC	AAAAATACATGTGGGGCGCG
	**pNbR1B12-P3**	CGGCGACAGATTTTTATCCCG	TTTGCTCTTCCTCCCACACA
	**pNbR1B12-P4**	GCTAAGGGTAAGGTCTGCGG	CTGTTCCACAACAATAGCAGCA
	**pNbR1B12-PH3**	TGTGTGGGAGGAAGAGCAAA	AACCTTCCCACGCCGAAAAA
	**pNbR1A6-P1**	GGAGCCCCTCATATCGAAGC	CCATGATATCCGGTTGGGCA
	**pNbR1A6-P2**	AGTGCAAGCTCGGAACTGAA	GCGAGTTGGCATCGATTGTG
	**pNbR1A6-P3**	CGGGTGGAATCGAGGGAAAA	TGATGCGCTCGGACGTAAAG
	**pNbR1A6-PH4**	TAATATCCGTGCCCAACCGG	TGGTATGCGTGTTACGATGTG
	**pNbR1B16-P1**	TGTAATGCTTGATGACAACGAACA	GGGTAAAGTAGGATCACTTTCTGC
	**pNbR1B16-P2**	TTGACCAACAAAGAGCTGGA	TGCTAAAGTCACAGTAGTTTTGAAA
	**pNbR1B16-P3**	ATCAGAGGCAGGATTTCCGC	GCTTAGTGGTCAAGGGGTTCA
	**pNbR1B16-P4**	TGATCATGGACCTCAGCAAGT	GGCAATAAGGGTGTGAAGCC
	**pNbR1B16-PH5**	GCATAGACAGGTCTGCAAGT	TCCTAGTTCATGCACCAAAACA
**Primers used for EMSA**	**WT^{CaR1B23AACAA} probe**	CTCTTTGAAACAAGAAGGGA	TCCCTTCTTGTTTCAAAGAG
	**M^{CaR1B23AACAA} probe**	CTCTTTGAAAAAAGAAGGGA	TCCCTTCTTTTTTCAAAGAG
	**WT^{CaR1B11AACAA} probe**	CGGGCTAGAACAAAACCGTC	GACGGTTTTGTTCTAGCCCG

	**$M^{CaR1B11AACAA}$ probe**	CGGGCTAGAAAAAAACCGTC	GACGGTTTTTTTCTAGCCCG
	**$WT^{CaR1AAACAA}$probe**	TGCGCTCCCTAACAAAGGAG	CTCCTTTGTTAGGGAGCGCA
	**$M^{CaR1AAACAA}$probe**	TGCGCTCCCTAAAAAAGGAG	CTCCTTTTTTAGGGAGCGCA
	**$WT^{CaR1B12AACAA}$ probe**	GAAAGAAATAACAAGAGTTA	TAACTCTTGTTATTTCTTTC
	**$MC^{aR1B12AACAA}$ probe**	GAAAGAAATAAAAAGAGTTA	TAACTCTTTTTATTTCTTTC
	**$WT^{CaR1A-6AACAA}$probe**	CTTTTTGGAACAACTCCAGT	ACTGGAGTTGTTCCAAAAAG
	**$M^{CaR1A-6AACAA}$ probe**	CTTTTTGGAAAAACTCCAGT	ACTGGAGTTTTTCCAAAAAG
	**$WT^{CaR1B16AACAA}$ probe**	TGAAAACAATAACAAGATCC	GGATCTTGTTATTGTTTTCA
	**$M^{CaR1B16AACAA}$ probe**	TGAAAAAAATAAAAAGATCC	GGATCTTTTTATTTTTTTCA
	**$WT^{CaR1B23HSE}$ probe**	CAATCCGTTCTAGAAAGTGA	TCACTTTCTAGAACGGATTG
	**$M^{CaR1B23HSE}$ probe**	CAATCCGAAAAAAAAAGTGA	TCACTTTTTTTTTCGGATTG
	**$WT^{CaR1B11HSE}$ probe**	CGTCATTTCTAGAAGTAGAT	ATCTACTTCTAGAAATGACG
	**$M^{CaR1B11HSE}$ probe**	CGTCATAAAAAAAAGTAGAT	ATCTACTTTTTTTTATGACG
	**$WT^{CaR1AHSE}$probe**	ATTACTTTCTAAGATTTATG	CATAAATCTTAGAAAGTAAT
	**$M^{CaR1AHSE}$probe**	ATTACTAAAAAAAATTTATG	CATAAATTTTTTTTAGTAAT
	**$WT^{CaR1B12HSE}$ probe**	CAAAGTTTCTAGAAAGAAAT	ATTTCTTTCTAGAAACTTTG
	**$M^{CaR1B12HSE}$ probe**	CAAAGTAAAAAAAAAGAAAT	ATTTCTTTTTTTTTACTTTG
	**$WT^{CaR1A-6HSE}$ probe**	AATGTTTTTTCTAGAATTAC	GTAATTCTAGAAAAAACATT
	**$M^{CaR1A-6HSE}$ probe**	AATGTTTTAAAAAAAATTAC	GTAATTTTTTTTAAAACATT
	**$WT^{CaR1B16HSE}$ probe**	GACCTTTCTAGAAAATCACT	AGTGATTTTCTAGAAAGGTC
	**$M^{CaR1B16HSE}$ probe**	GACCTAAAAAAAAAATCACT	AGTGATTTTTTTTTTAGGTC
	**3x AACAA probe**	AACAA AACAA AACAA	TTGTT TTGTT TTGTT
	**3x AACAAm probe**	AAAAA AAAAA AAAAA	TTTTT TTTTT TTTTT
	**3x HSE probe**	AATTCTAGAAAAAATTCTAGAAAAAATTCTAGAAAA	TTTTCTAGAATT TTTTCTAGAATT TTTTCTAGAATT
	**3x HSEm probe**	AATTCTTTCTAA AATTCTTTCTAA AATTCTTTCTAA	TTAGAAAGAATT TTAGAAAGAATT TTAGAAAGAATT

**Primers used for NLRs VIGS analysis**	***CaR1B23*-VIGS**	GGGGACAAGTTTGTACAAAAAAGCAGGCTTC TGTGGGAAGCTGATGTGTGG	GGGGACCACTTTGTACAAGAAAGCTGGGTCTCTTCTCCATCTCGGCCAGA
	***CaR1B11*-VIGS**	GGGGACAAGTTTGTACAAAAAAGCAGGCTTC ACCACTTCCCTCCAACGTTG	GGGGACCACTTTGTACAAGAAAGCTGGGTCTCCTCGCTCCGATCATTTGG
	***CaR1A*-VIGS**	GGGGACAAGTTTGTACAAAAAAGCAGGCTTC AGCTACGACATGTGGACACC	GGGGACCACTTTGTACAAGAAAGCTGGGTCCAGGCTCGAAGGGAAGTGAA
	***CaR1B12*-VIGS**	GGGGACAAGTTTGTACAAAAAAGCAGGCTTC GTCCTTTGGAGCTACGCAGT	GGGGACCACTTTGTACAAGAAAGCTGGGTCCGGCACAAACTCCTCAGCTA
	***CaR1A6*-VIGS**	GGGGACAAGTTTGTACAAAAAAGCAGGCTTC GGCATGCAGCTGACCAAAAT	GGGGACCACTTTGTACAAGAAAGCTGGGTCTCCCAAGCCTCAGTACTCCA
	***CaR1B16*-VIGS**	GGGGACAAGTTTGTACAAAAAAGCAGGCTTC GGACGCATTCCTCGCAAATC	GGGGACCACTTTGTACAAGAAAGCTGGGTCAACGTCCCATTTTCCCTGCT
	***NbR1B23*-VIGS**	GGGGACAAGTTTGTACAAAAAAGCAGGCTTCCCTGCCCCAGTACGTTGATT	GGGGACCACTTTGTACAAGAAAGCTGGGTCACGGCAGGTGACAGCTAAAA
	***NbR1B11*-VIGS**	GGGGACAAGTTTGTACAAAAAAGCAGGCTTCCAAGTCAGCAGAGGCCGTTA	GGGGACCACTTTGTACAAGAAAGCTGGGTCCGCCCTGCTAGCAATCCATA
	***NbR1B12*-VIGS**	GGGGACAAGTTTGTACAAAAAAGCAGGCTTCGAACTCCTACTCCCGCGAAG	GGGGACCACTTTGTACAAGAAAGCTGGGTCTCAGCCATGCTATGACCACC
	***NbR1A6*-VIGS**	GGGGACAAGTTTGTACAAAAAAGCAGGCTTCGAAGATATGGTTGCGCTGGC	GGGGACCACTTTGTACAAGAAAGCTGGGTCCACTAGTGACGGCTGCAGAA
	***NbR1B16*-VIGS**	GGGGACAAGTTTGTACAAAAAAGCAGGCTTCGGACGCATTCCTCGCAAATC	GGGGACCACTTTGTACAAGAAAGCTGGGTCCCTTGAGGGTCGGAAACTCC
**Primers used for promoter analysis**	**pCaR1B23-LUC**	GTCGACGGTATCGATAAGCTTAGCTAACGAATGCACGACTT	CAGGAATTCGATATCAAGCTTCACGGCTGTGGATCTCCGAG
	**pCaR1B11-LUC**	GTCGACGGTATCGATAAGCTTATTCATATAAAGGACCTCAA	CAGGAATTCGATATCAAGCTTCCATCAGAATTGTCATCACT
	**pCaR1A-LUC**	GTCGACGGTATCGATAAGCTTAGTGGATGAGTTTGGGGTGG	CAGGAATTCGATATCAAGCTTAGAATCGGGAGATCACTACC
	**pCaR1B12-LUC**	GTCGACGGTATCGATAAGCTTTGAGTTGGATATATAGTTTC	CAGGAATTCGATATCAAGCTTACGTGACTTCCGAGCAGTAG
	**pCaR1A6-LUC**	GTCGACGGTATCGATAAGCTTTTCAAGTATGGTGCCCCATG	CAGGAATTCGATATCAAGCTTCACCTCCTTCCTGAGATAAC
	**pCaR1B16-LUC**	GTCGACGGTATCGATAAGCTTTCTCAATTCTCACCACCACC	CAGGAATTCGATATCAAGCTTGTTCACTGAATTTGACGTAT

	**pCaR1B23-GW**	GGGGACAAGTTTGTACAAAAAAGCAGGCTTCAGCTAACGAATGCACGACTT	GGGGACCACTTTGTACAAGAAAGCTGGGTCCACGGCTGTGGATCTCCGAG
	**pCaR1B11-GW**	GGGGACAAGTTTGTACAAAAAAGCAGGCTTCATTCATATAAAGGACCTCAA	GGGGACCACTTTGTACAAGAAAGCTGGGTCCCATCAGAATTGTCATCACT
	**pCaR1A-GW**	GGGGACAAGTTTGTACAAAAAAGCAGGCTTCAGTGGATGAGTTTGGGGTGG	GGGGACCACTTTGTACAAGAAAGCTGGGTCAGAATCGGGAGATCACTACC
	**pCaR1B12-GW**	GGGGACAAGTTTGTACAAAAAAGCAGGCTTCTGAGTTGGATATATAGTTTC	GGGGACCACTTTGTACAAGAAAGCTGGGTCACGTGACTTCCGAGCAGTAG
	**pCaR1A6-GW**	GGGGACAAGTTTGTACAAAAAAGCAGGCTTCTTCAAGTATGGTGCCCCATG	GGGGACCACTTTGTACAAGAAAGCTGGGTCCACCTCCTTCCTGAGATAAC
	**pCaR1B16-GW**	GGGGACAAGTTTGTACAAAAAAGCAGGCTTCTCTCAATTCTCACCACCACC	GGGGACCACTTTGTACAAGAAAGCTGGGTCGTTCACTGAATTTGACGTAT

Supplementary Data Table S2. Grading standards for evaluation of disease resistance of pepper plants to *R. solanacearum* by root irrigation[3]

**Score**	**Condition**
0	Pepper plant is normal and asymptomatic.
1	Plant has slight withering, the basal one or two leaves are withered, but the apical region of the plant is normal.
2	In addition to the top leaves, one or two leaves are withered, but the apical region of the plant is normal.
3	Two-thirds of the leaves of the pepper plant are withered, while the top of the plant is normal.
4	The whole plant is withered or dead.

Figures

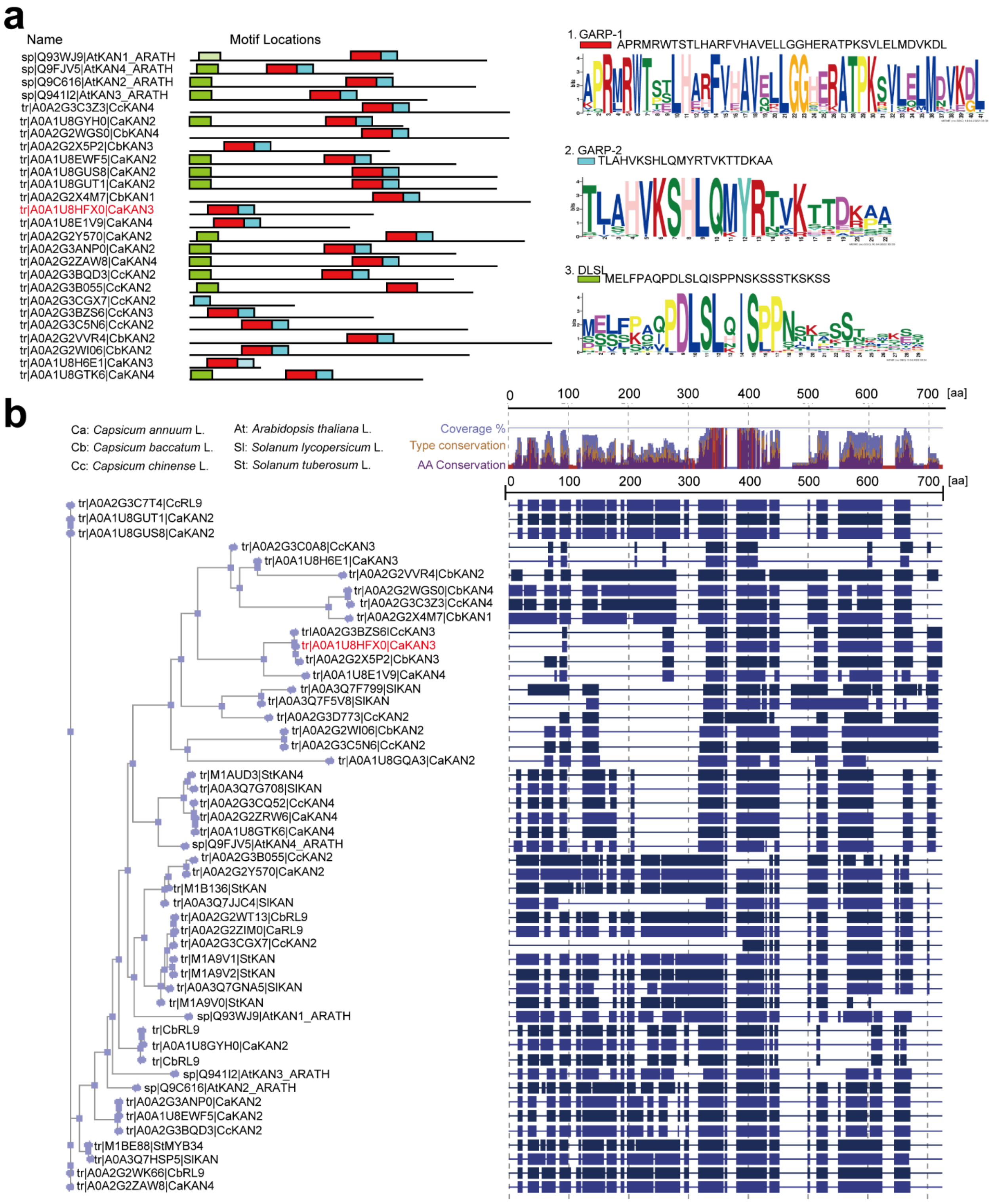

Supplementary Data Fig.1|CaKAN3 sequence analysis and phylogenetic tree of CaKAN3 and the KANADI members in pepper, potato, tomato and Arabidopsis. a, The hidden Markov model (HMM) profile of the GARP domain (Pfam: PF16731) (http://pfam.xfam.org/)

was used as a BLAST query against the pepper genome database PGP (http://peppergenome.snu.ac.kr/) and the KANADI proteins in *Arabidopsis*, *Solanum lycopersicum* and *Solanum tuberosum* from UniProt (https://www.uniprot.org/). Motifs were identified by MEME tools in KANADIs. In total, 3 motifs were identified, and only the GARP domain was identified in CaKAN3. **b**,Phylogenetic tree of KANADI members in pepper, potato, tomato and Arabidopsis; the phylogenetic tree was constructed with the Tree Browser from Solgenomics (https://solgenomics.net/tools/tree_browser).

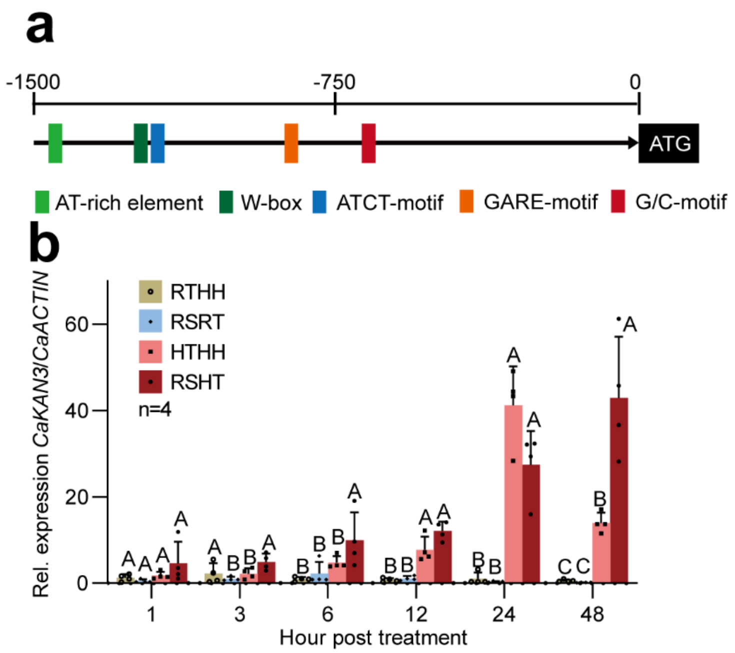

Supplementary Data Fig.2| Response of *CaKAN3* to *Ralstonia solanacearum* infection under room temperature (RSRT), high-temperature and high-humidity (HTHH) stress and *Ralstonia solanacearum* infection under high temperature and high humidity (RSHT). a, Cis-elements in the promoter region of *CaKAN3* by plant care (http://bioinformatics.psb.ugent.be/webtools/plantcare/html/). **b**, Transcript levels of *CaKAN3* in the roots of pepper plants challenged with RSRT, HTHH or RSHT, its transcript levels under RTHH (room temperature and high humidity) at 1 hpt were set to 1, and the data are shown as the means ± standard errors of four replicates. Different uppercase letters above the bars indicate significant differences between means ($P < 0.01$) by Fisher's protected LSD test. Source data are provided as a Source Data file.

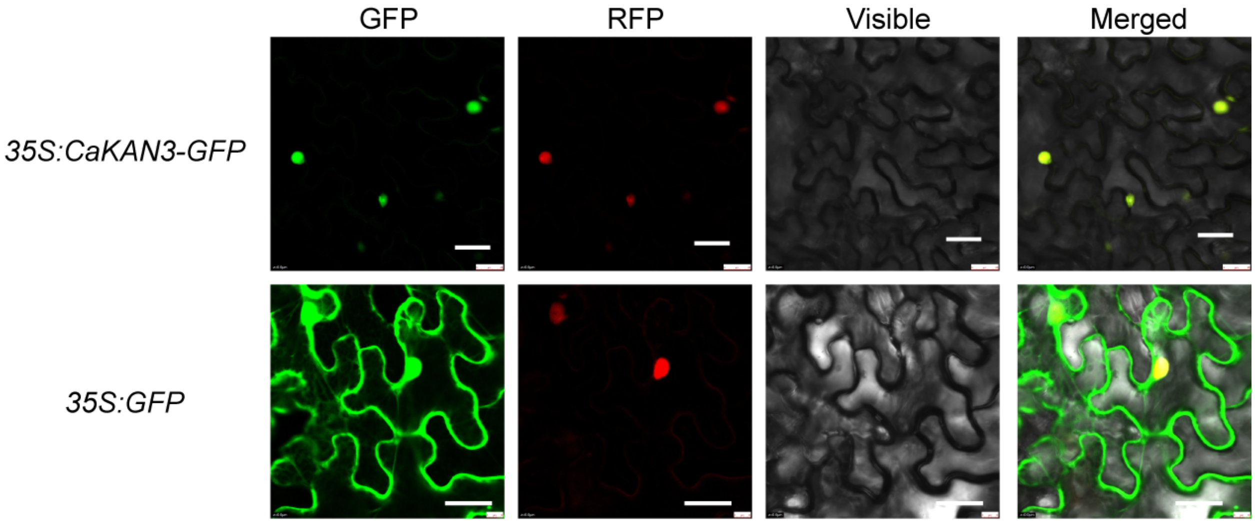

Supplementary Data Fig.3|Nuclear Localization of CaKAN3 in *N. benthamiana* Epidermal Cells. *N. benthamiana* leaves were infiltrated with Agrobacterium GV3101 cells containing *35Spro:CaKAN3-GFP* (using *35Spro:GFP* as a control). NbH2B (histone H2B)-RFP was used to indicate the nucleus. Subcellular localization of the CaKAN3-GFP fusion protein or control GFP was captured on a fluorescence confocal microscope at 24 hpi. Fluorescence images (left), bright-field images (middle), and the corresponding overlay images (right) of representative cells expressing GFP or CaKAN3-GFP fusion protein are shown, Bars = 50 μm. The experiment was carried out thrice with similar results.

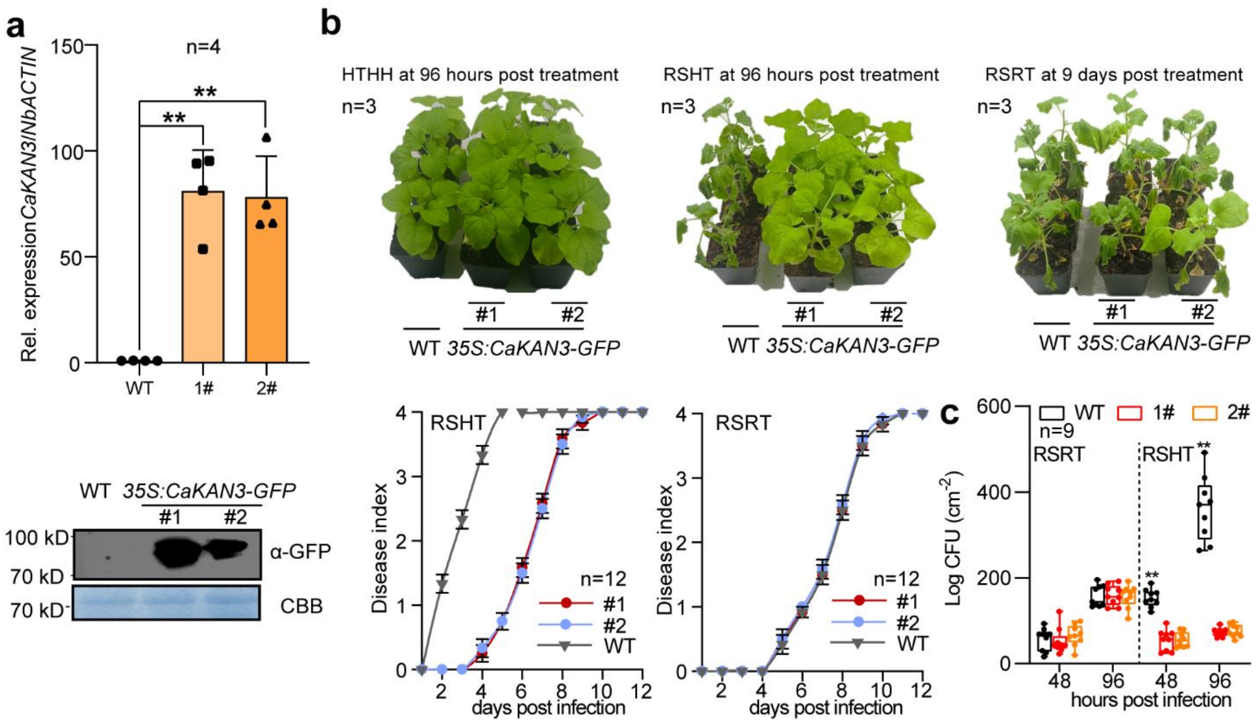

Supplementary Data Fig.4|The effect of CaKAN3ectopicoverexpression on the response of *Nicotiana benthamiana* plants to *Ralstonia solanacearum* infection under room temperature (RSRT), hightemperature and high humidity (HTHH) or *Ralstonia solanacearum* infection under hightemperature and highhumidity (RSHT). a, Confirmation of *CaKAN3* overexpression by RT–qPCR and immunoblot analysis with antibody of GFP in the two T_2 transgenic *N. benthamiana* lines. α-GFP: anti body of GFP. **b**, The *CaKAN3*-overexpressing *N. benthamiana* plants displayed lower level of disease index and higher level of resistance to RSHT but not to RSRT compared to the wild-type control plants. The experiment was carried out twice with similar results. **c**, *R. solanacearum*-inoculated, *CaKAN3*-overexpressing *N. benthamiana* plants showed higher pathogen growth than the wild type (shown as colony-forming units [cfu]) upon RSHT but not upon RSRT. Data are shown as the means ± standard errors of four replicates. Asterisks above the bars indicate significant differences between means ($P< 0.01$) by Fisher's protected t test. The center line represents the median value and the boundaries indicate the 25th percentile (upper) and the 75th percentile (lower). Whiskers extend to the largest and smallest value. In **b**, and **c**, the experiments were carried out three with similar results. In **a**, to **c**, source data are provided as a Source Data file.

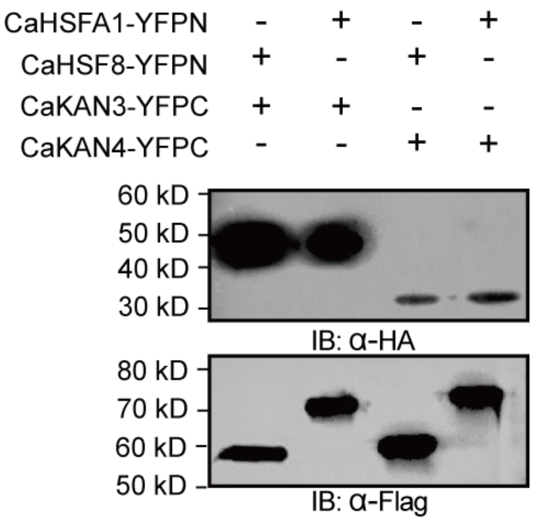

Supplementary Data Fig.5|The success of transient overexpression of CaHSFA1,CaHSF8, CaKAN3 and CaKAN4 in *N. benthamiana* plants. The proteins were isolated from leaves of *N. benthamiana* plants agroinfiltrated with GV3101 cells at 48 hpi. The experiment was carried out once.

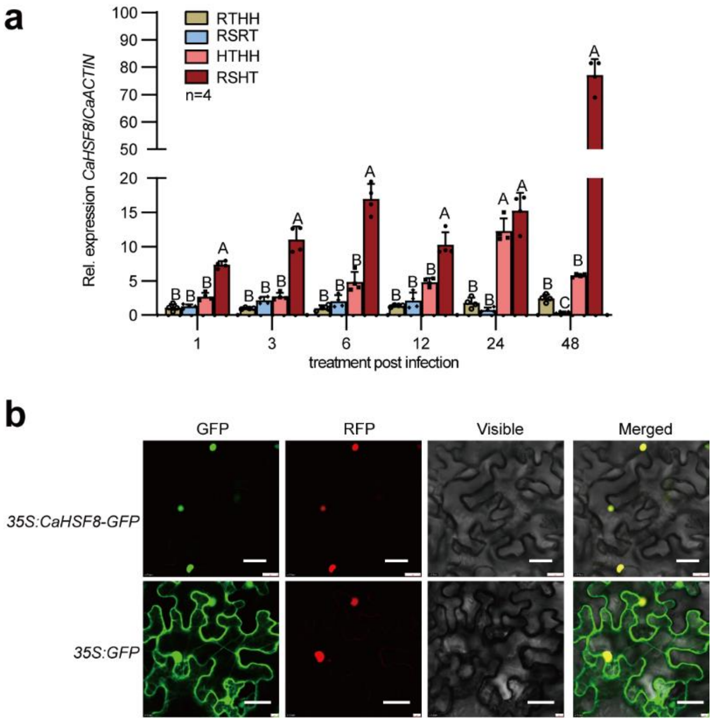

Supplementary Data Fig.6|Transcript expression level analysis and subcellular localization analysis of CaHSF8. a, Transcript levels of *CaHSF8* in the roots of pepper plants challenged with RSRT, HTHH or RSHT. The transcript levels of *CaHSF8* under RTHH at 1 hpt were set to 1, and the data are shown as the means ± standard errors of four replicates. Different uppercase letters above the bars indicate significant differences between means ($P < 0.01$) by Fisher's protected LSD test. Source data are provided as a Source Data file. **b**, Subcellular localization of CaHSF8-GFP in epidermal cells of *N. benthamiana* leaves. NbH2B (histone H2B)-RFP was used to indicate the nucleus. Red and green fluorescence, visible light and merged images were taken on a confocal microscope. Bars = 50 μm.

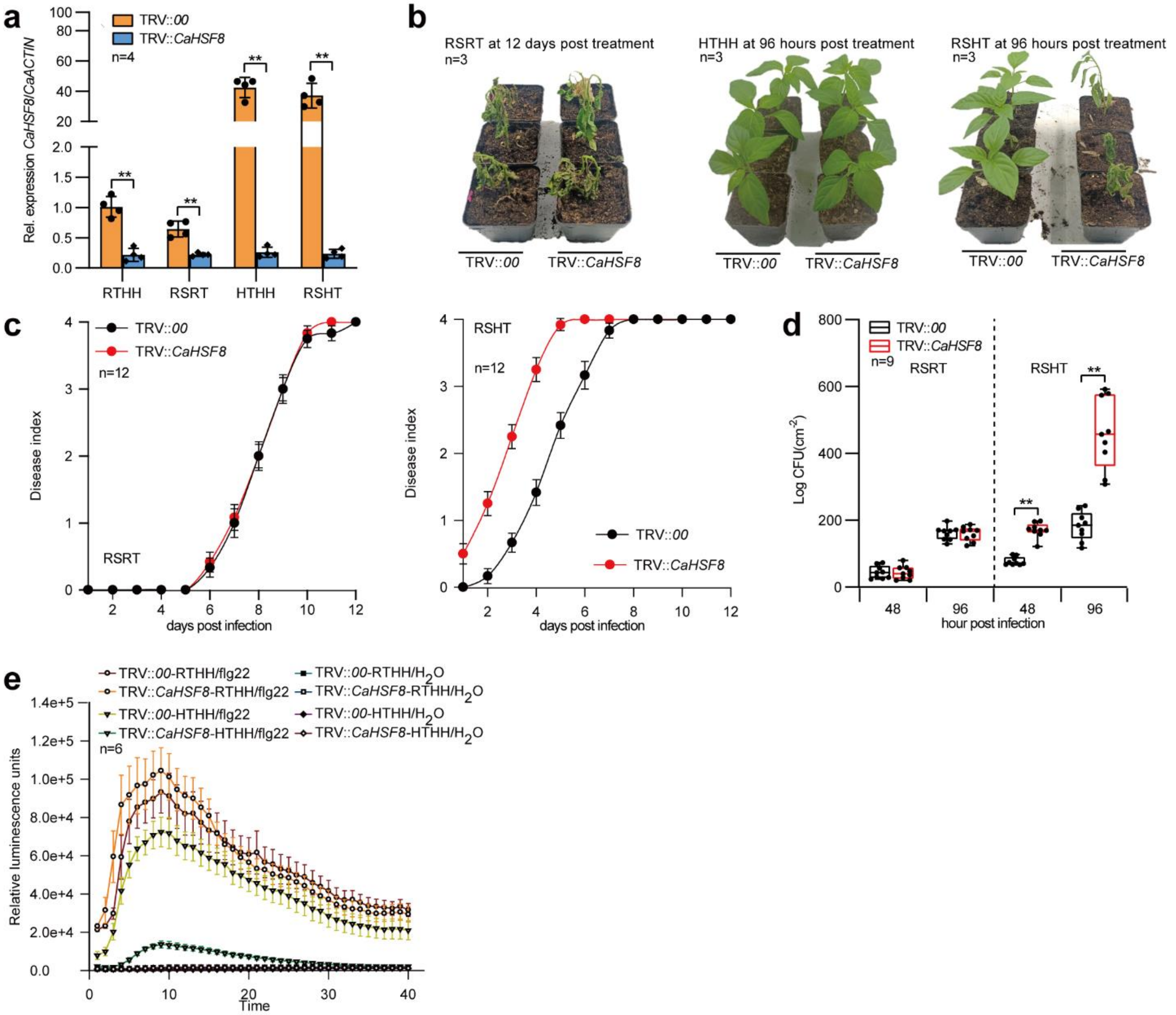

Supplementary Data Fig.7|The silencing of CaHSF8 significantly reduced pepper immunity against RSHT. a, The success of *CaHSF8* silencing by virus-induced gene silencing (VIGS) was determined by measuring the transcript levels of *CaHSF8* in RTHH-, RSRT-, HTHH- and RSHT-challenged TRV:*CaHSF8* pepper plants at 24 hours post-treatment (hpt). The transcript levels of *CaHSF8* in TRV:*00* pepper plants under RTHH were set to 1. **b**, Effect of *CaHSF8* silencing on the response of pepper plants to RSRT and RSHT treatment at 3 and 12 dpt, respectively. **c**, and **d**, The growth of *R. solanacearum* in *R. solanacearum*-inoculated *CaHSF8*-silencing plants under RSHH or upon HTHH, shown as colony-forming units (cfu). Data were shown as the means ±standard errors of eight replicates. Asterisks above the bars indicate significant differences between means ($P< 0.01$), as calculated with a t test. The center line represents the median value and the boundaries indicate the 25th percentile (upper) and the 75th percentile (lower). Whiskers extend to the largest and smallest value. **e**, Decreased flg22-induced H_2O_2 production in *CaHSF8*-silencingpepper plants upon HTHH. The results shown were representative of two independent experiments. Data were shown as the means ±standard errors

of six replicates. In **b**, and **c**, the experiments were carried out three with similar results. In **a**, **c**, **d**, and **e**, source data are provided as a Source Data file.

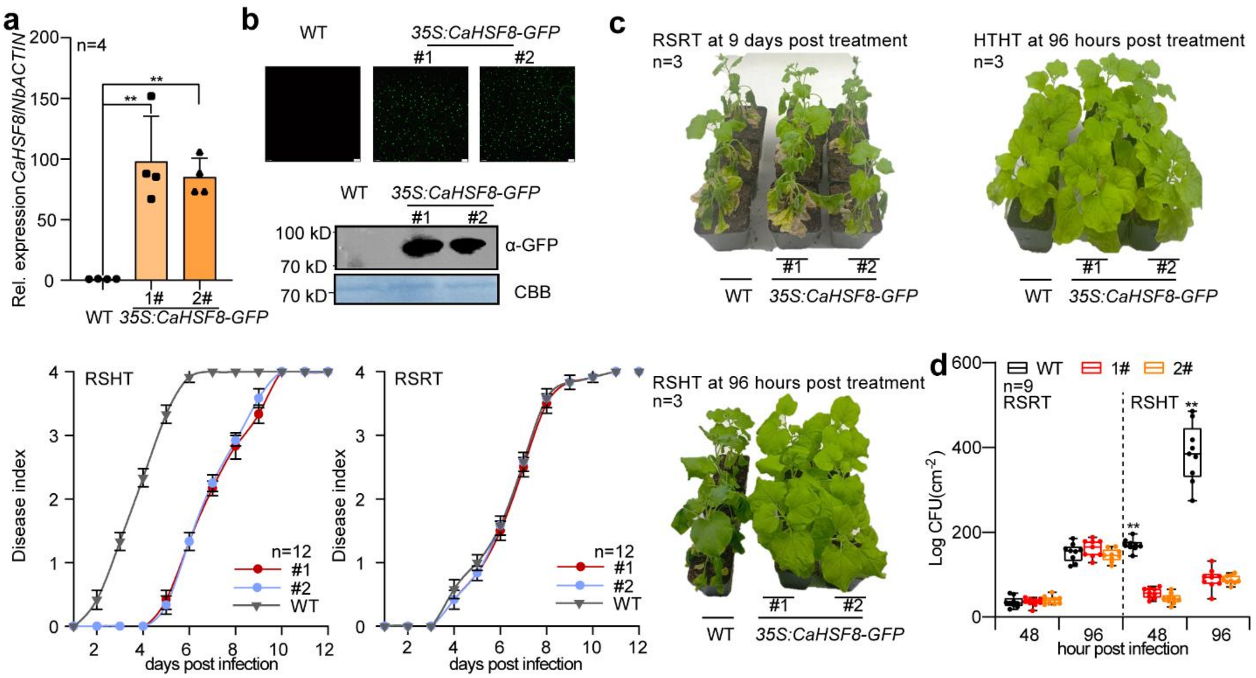

Supplementary Data Fig.8|The overexpression of CaHSF8 significantly potentiated *N. benthamiana* immunity against RSHT. a, and **b**, Confirmation of *CaHSF8* overexpression by fluorescence detection, RT–qPCR and immunoblot analysis with antibody of GFP in the two T_2 transgenic *N. benthamiana* lines. α-GFP: antibody of GFP. **c**, The *CaHSF8*-overexpressing *N. benthamiana* plants displayed lower level of disease index and higher level of resistance upon RSHT but not upon RSRT than the wild-type control plants. The experiment was carried out twice with similar results. **d**, *R. solanacearum*-inoculated *CaHSF8*-overexpressing *N. benthamiana* plants showed higher pathogen growth than the wild type (shown as colony-forming units [cfu]) upon RSHT but not upon RSRT. Data were shown as the means ± standard errors of four replicates. The center line represents the median value and the boundaries indicate the 25th percentile (upper) and the 75th percentile (lower). Whiskers extend to the largest and smallest value. In **a** and **d**, the asterisk above the bars indicates significant differences between means (P< 0.01) by Fisher's protected t test. In **a**, **c**, and **d**, source data are provided as a Source Data file.

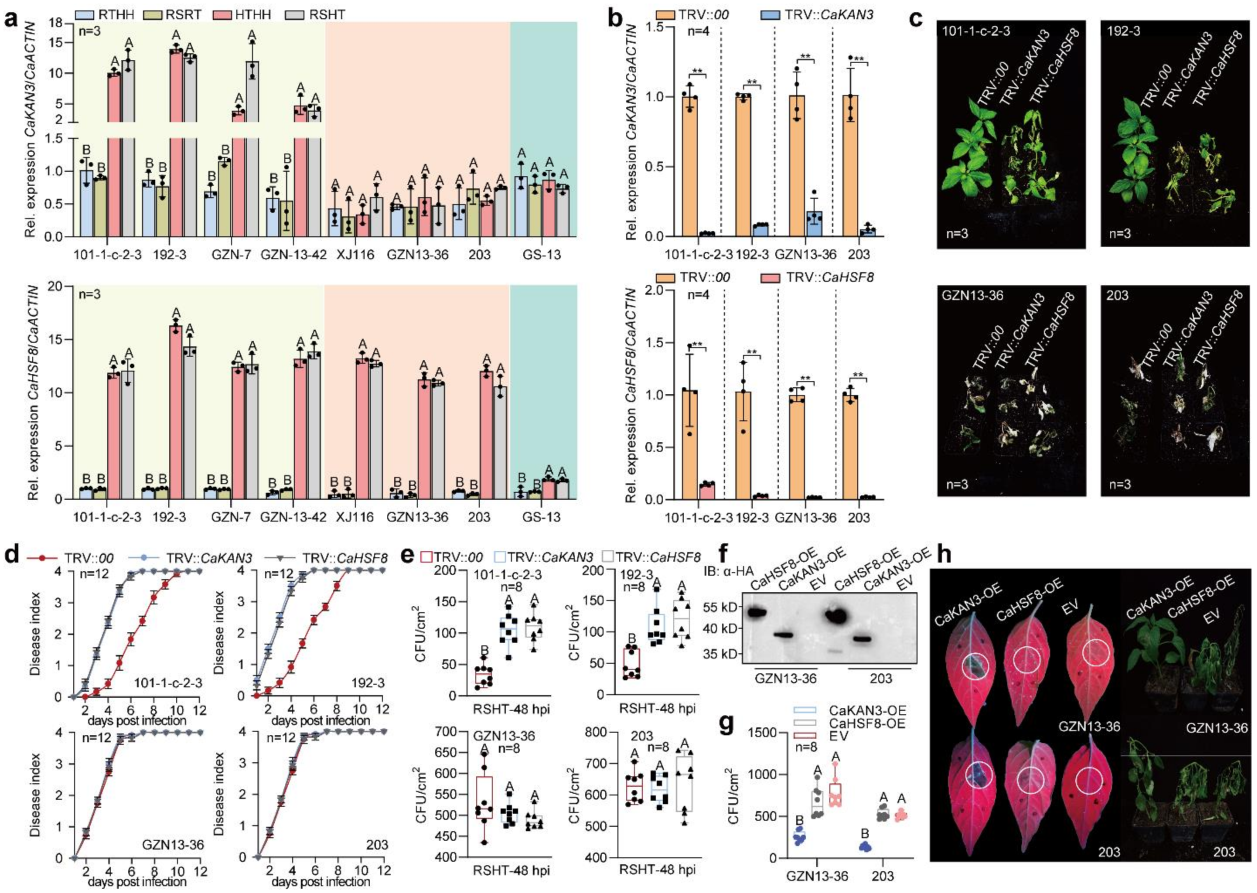

Supplementary Data Fig.9|The expression of *CaHSF8* and *CaKAN3* and their functions in immunity of pepper inbred lines against RSHT. a, The relative transcript level of *CaKAN3* and *CaHSF8* in pepper inbred lines with different level of resistance to RSHT in pepper plants upon RTHH, RSRT, HTHH or RSHT. Data are shown as the means ± standard errors of three replicates. The transcript levels of 101-1-c-2-3/RTHH were set to 1. **b**, The silencing efficiency of *CaKAN3* and *CaHSF8* in different pepper inbred lines by RT-qPCR. The transcript levels of *CaKAN3* and *CaHSF8* in TRV::*00* pepper plants under RTHH were set to 1. Data are shown as the means ± standard errors of four replicates. Asterisks above the bars indicate significant differences between means (P< 0.01) by Fisher's protected t test. **c**, The silencing of *CaKAN3* or *CaHSF8* significantly reduced pepper immunity against RSHT in pepper lines with higher level of RSHT resistance but not in lines with lower level of RSHT resistance. **d**, The silencing of *CaKAN3*or *CaHSF8*significantly increased dynamic disease index from 0 to 12 dpt in pepper lines with high level of RSHT resistance but not in lines with lower level of RSHT resistance. Data were shown as the means ± standard errors of twelve replicates. **e**, The silencing of *CaHSF8*or *CaKAN3*supported an enhanced level of bacterial growth at 48 hpi by leaf inoculation in pepper lines with high level of RSHT resistance but not in lines with lower level of RSHT resistance. **f**, The success of transient overexpression of CaHSF8-HA or CaKAN3-HA by immune blotting using antibody of HA in pepper line GZN13-36 and 203 that are susceptible to RSHT. The experiment was carried out once. **g**, The transient overexpression of CaKAN3 but not CaHSF8 significantly repressed the bacterial growth in the RSHT susceptible line GZN13-36

and 203. **h**, The transient overexpression of CaKAN3 significantly repressed the proliferation of RS by activating HR response in leaves of RSHT susceptible line GZN13-36 and 203 at 48 hpi, and conferred enhanced RSHT resistance at 6 dpi, but the transient overexpression of *CaHSF8* did not make any difference. In **a**, **e**, and **g**, Different uppercase letters above the bars indicate significant differences between means ($P < 0.01$) by Fisher's protected LSD test. In **c**, **d**, and **h**, the experiments were carried out three with similar results. In **e** and **g**, the center line represents the median value and the boundaries indicate the 25th percentile (upper) and the 75th percentile (lower). Whiskers extend to the largest and smallest value. In **a**, **b**, **d**, **e**, and **g**, source data are provided as a Source Data file.

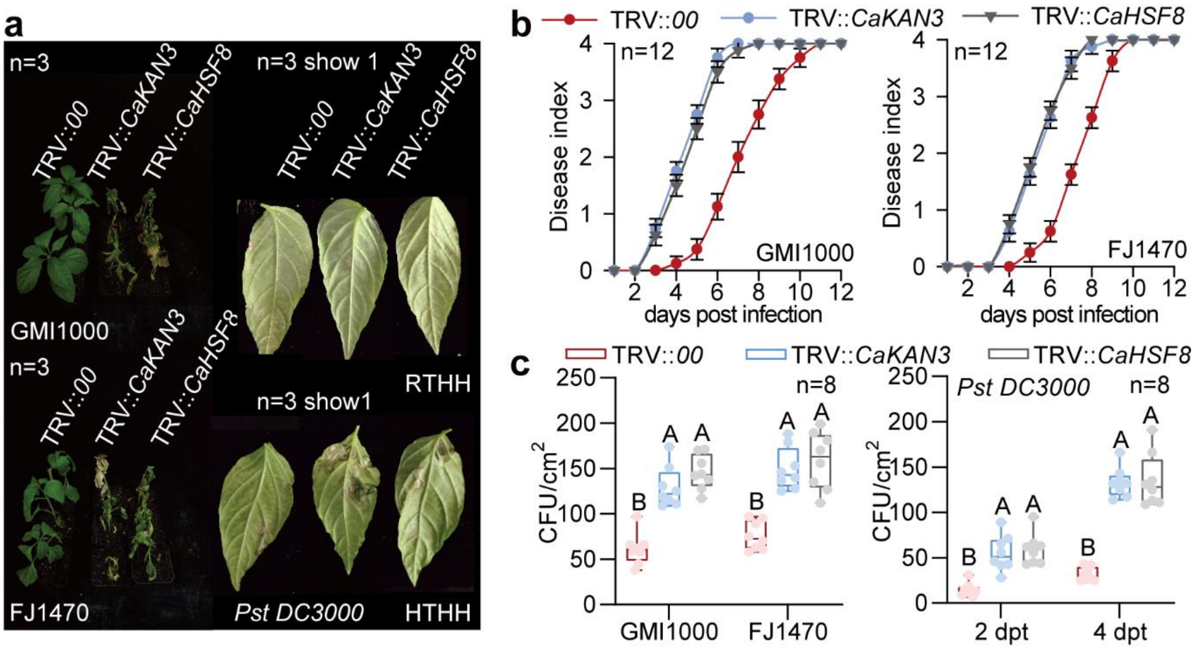

Supplementary Data Fig.10|The effect of *CaKAN3* or *CaHSF8* silencing on the response of pepper plants to inoculation of different *Ralstonia solanacearum* strains(FJ1470,GMI1000) and to *Pst DC3000*. **a**, The silencing of *CaKAN3* or *CaHSF8* significantly increased susceptibility of pepper plants to inoculation of both FJ1470 and GMI1000 or *Pst DC3000* under HTHH, but did not affect the response of pepper plant to the inoculation of *Pst DC3000* under RTHH. **b**, The silencing of *CaKAN3* and *CaHSF8* significantly increased the dynamic disease index of RSHT challenged pepper plants from 0 to 12 dpt. Data were shown as the means ± standard errors of twelve replicates. **c**, The silencing of *CaKAN3* or *CaHSF8* promoted the *Ralstonia solanacearum* or *Pst DC3000* growth displayed by cfu(clone forming units) in the leaves of pepper plants inoculated using GMI1000,FJ1470 at 48 hpt or *Pst DC3000* at 2 and 4dpt.Different uppercase letters above the bars indicate significant differences between means ($P < 0.01$) by Fisher's protected LSD test. The center line represents the median value and the boundaries indicate the 25th percentile (upper) and the 75th percentile (lower). Whiskers extend to the largest and smallest value. In **a**, and **b**, the experiments were carried out three with similar results. In **b**, and **c**, source data are provided as a Source Data file.

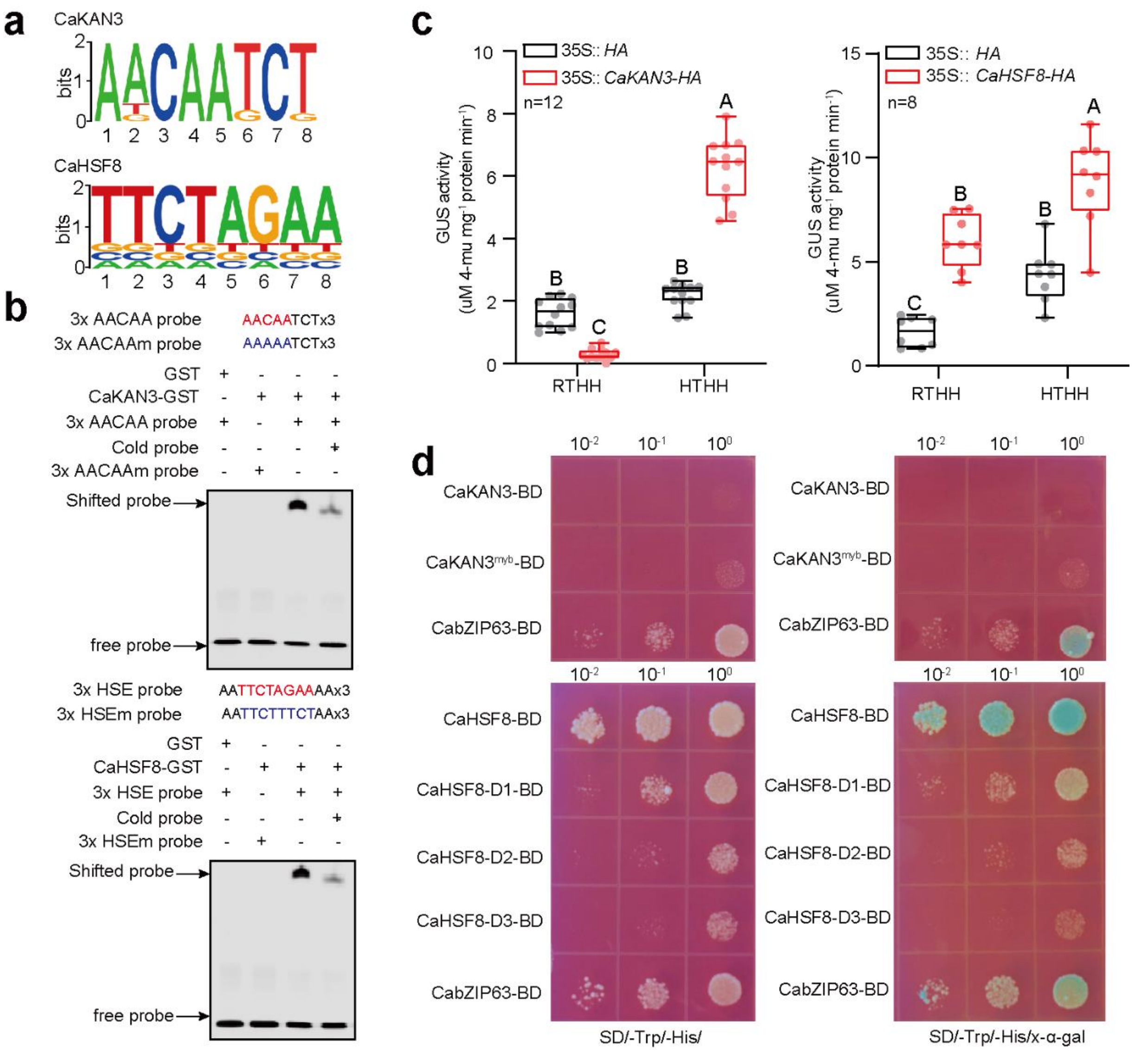

Supplementary Data Fig.11| The possible CaKAN3- or CaHSF8-bound cis-elements were determined by ChIP-seq and the transcriptional activity of CaKAN3 or CaHSF8. a, The peak measured by ChIP-seq and its matched cis-element by HOMER motif search. **b**, The binding of CaKAN3-GST or CaHSF8-GST to the 3×AACAA or 3×HSE cis-element fragment by electrophoretic mobility shift assay in vitro. The experiment was carried out once. **c**, Activity of GUS driven by synthetic promoters containing AACAA or HSE in leaves of transient overexpression of CaKAN3 or CaHSF8 or control pepper plants at 48 hpt. Data represent the mean ± SD of twelve biological replicates. ifferent uppercase letters above the bars indicate significant differences between means ($P< 0.01$) by Fisher's protected LSD test. Source data are provided as a Source Data file. The center line represents the median value and the boundaries indicate the 25th percentile (upper) and the 75th percentile (lower). Whiskers extend to the largest and smallest value. **d**, Transcriptional activity of *CaKAN3* or *CaHSF8* by nutrition disfigurement assay in GAL4-based yeast one-hybrid system. In **c**, and **d**, the experiments were carried out twice with similar results.

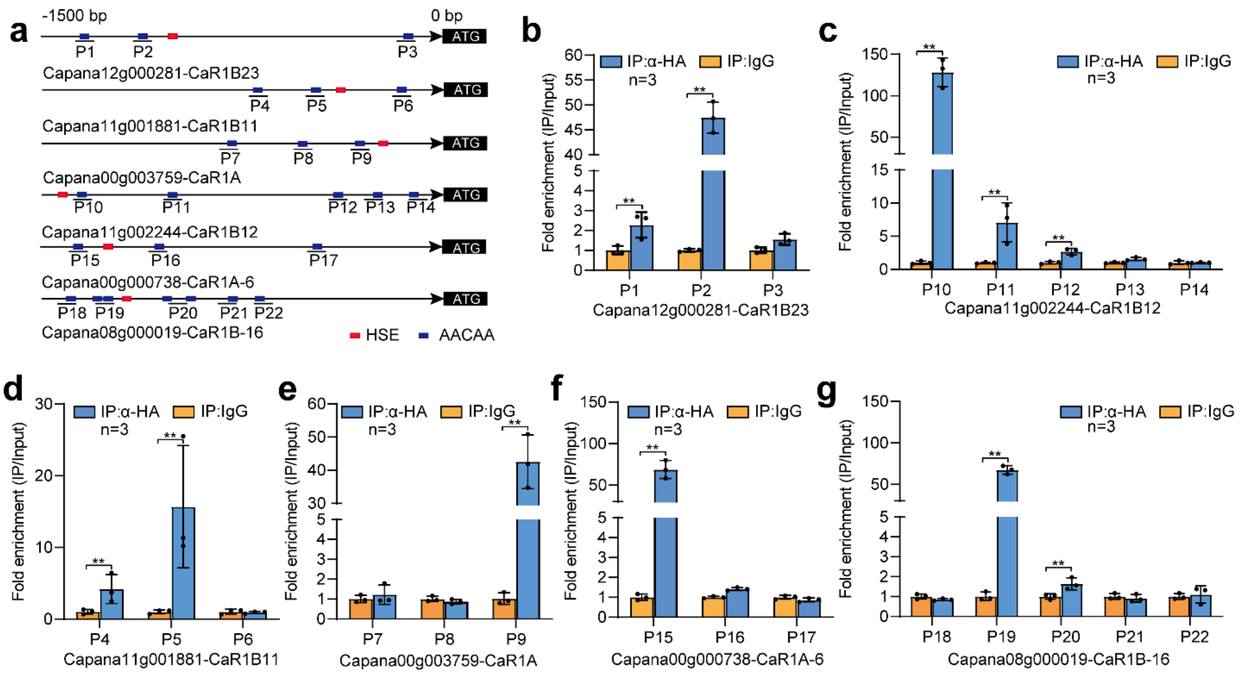

Supplementary Data Fig.12|Bindings of CaKAN3 to the promoters of *CaR1B23*, *CaR1B12*, *CaR1A*, *CaR1B11*, *CaR1A6* and *CaR1B16* by ChIP-qPCR. a, Schematic diagram of the cis-element HSE (red squares) and AACAA (blue squares)in the promoters of *CaR1B23*, *CaR1B12*, *CaR1A*, *CaR1B11*, *CaR1A6* and *CaR1B16*. P1-P22 were fragments for designing the primer pairs used to amplify the target DNA fragments in the promoters. **b**, to **g**, The bindings of CaKAN3 to the different cis-elements by ChIP-qPCR,GV3101 cells containing *35S:CaKAN3-HA* were infiltrated into pepper leaves, which were harvested at 48 hpi for ChIP–qPCR analysis using specific primer pairs; IP: IgG was used as the control. The enrichment levels of the tested genes were compared with those in the control, and the relative enrichment of IgG was set to a value of 1 after normalization by input. Data represent the mean ± SD of three biological replicates. Different capital letters above the bars indicate significant differences (P< 0.01) by Fisher's protected t test. In **b**, to **g**, source data are provided as a Source Data file.

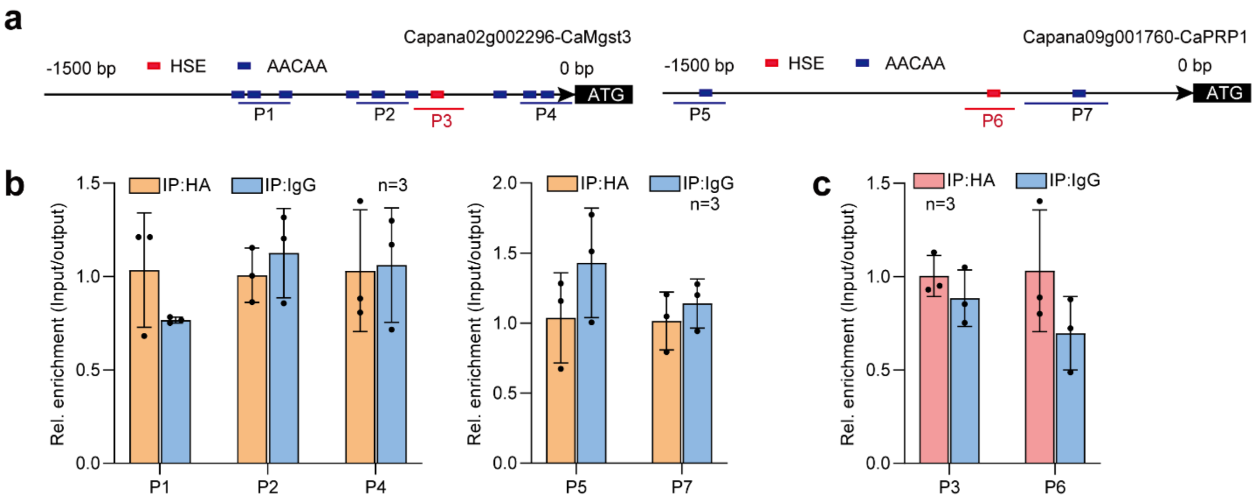

Supplementary Data Fig.13|Neither CaKAN3 nor CaHSF8 bound the promoters of *CaMgst3* and *CaPRP1*. **a**, The distribution of the fragments in the promoter of CaMgst3 or CaPRP1 corresponding to primers used in ChIP-qPCR to assay the enrichment of CaKAN3 or CaHSF8 in the promoters of *CaMgst3* and *CaPRP1*. **b**, The enrichment of CaKAN3 in the promoter of CaMgst3 or CaPRP1 by ChIP-qPCR. **c**, The enrichment of CaHSF8 in the promoter of *CaMgst3* and *CaPRP1* by ChIP-qPCR. **b**, and **c**, GV3101 cells containing *35S:CaKAN3-HA* or *35S:CaHSF8-HA* were infiltrated into pepper leaves, which were harvested at 48 hpi for ChIP-qPCR analysis using specific primer pairs; IP: IgG was used as the control. The enrichment levels of the tested genes were compared with those in the control, and the relative enrichment of anti-HA was set to a value of 1 after normalization by input. Data represent the mean ± SD of three biological replicates. In **b**, to **c**, source data are provided as a Source Data file.

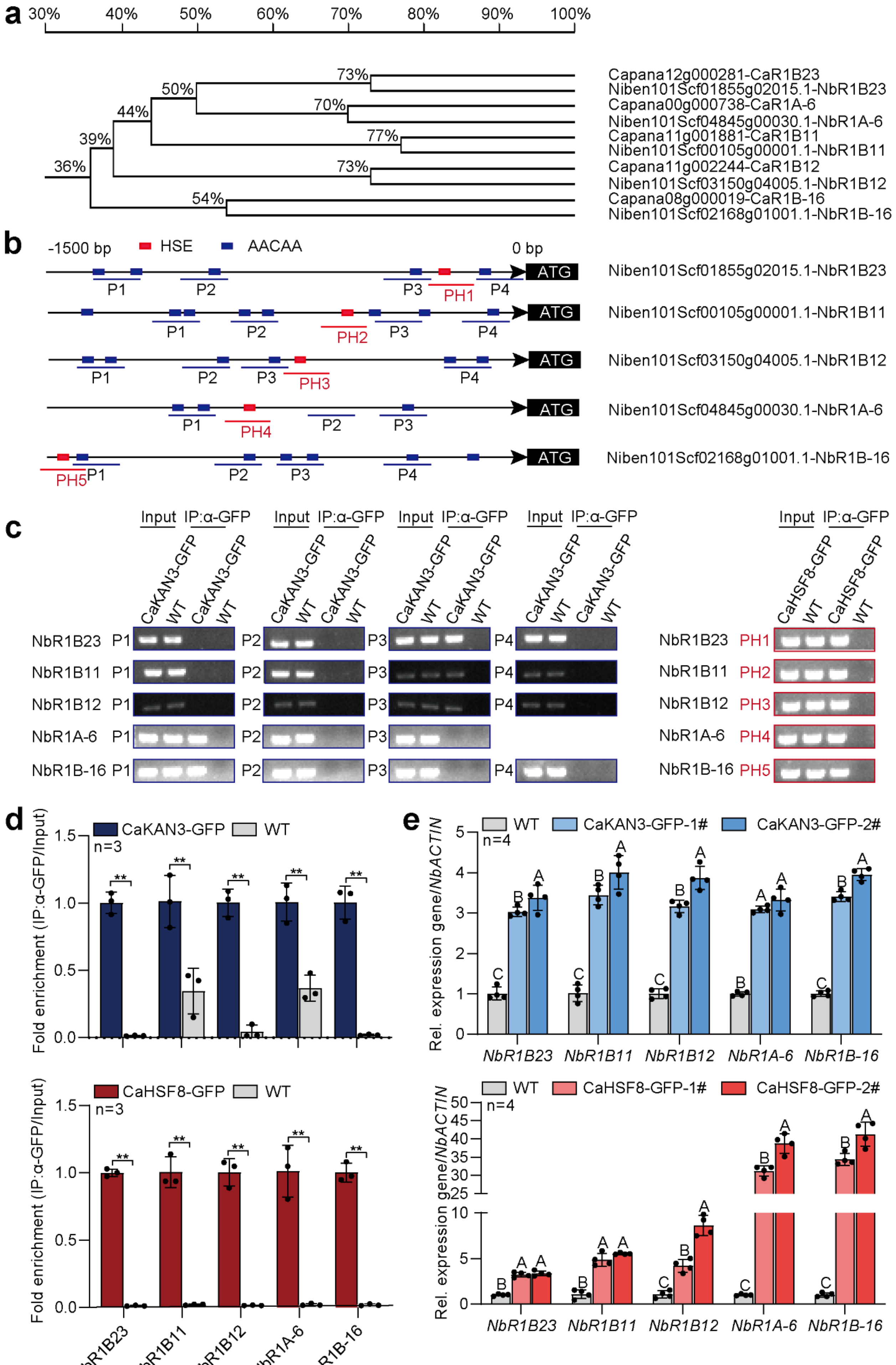
a
30%
40%
50%
60%
70%
80%
90%
100%
73%
50%
70%
44%
77%
39%
73%
36%
54%
Capana12g000281-CaR1B23
Niben101Scf01855g02015.1-NbR1B23
Capana00g000738-CaR1A-6
Niben101Scf04845g00030.1-NbR1A-6
Capana11g001881-CaR1B11
Niben101Scf00105g00001.1-NbR1B11
Capana11g002244-CaR1B12
Niben101Scf03150g04005.1-NbR1B12
Capana08g000019-CaR1B-16
Niben101Scf02168g01001.1-NbR1B-16
b
-1500 bp
HSE
AACAA
0 bp
ATG
P1
P2
P3
P4
PH1
PH2
PH3
PH4
PH5
Niben101Scf01855g02015.1-NbR1B23
Niben101Scf00105g00001.1-NbR1B11
Niben101Scf03150g04005.1-NbR1B12
Niben101Scf04845g00030.1-NbR1A-6
Niben101Scf02168g01001.1-NbR1B-16
c
Input
IP:α-GFP
CaKAN3-GFP
WT
CaHSF8-GFP
NbR1B23
NbR1B11
NbR1B12
NbR1A-6
NbR1B-16
d
CaKAN3-GFP
WT
n=3
**
Fold enrichment (IP:α-GFP/Input)
1.5
1.0
0.5
0.0
CaHSF8-GFP
e
WT
CaKAN3-GFP-1#
CaKAN3-GFP-2#
n=4
Rel. expression gene/NbACTIN
NbR1B23
NbR1B11
NbR1B12
NbR1A-6
NbR1B-16
CaHSF8-GFP-1#
CaHSF8-GFP-2#
A
B
C

Supplementary Data Fig.14|The targeting of NLR genes in *N. benthamiana* by CaKAN3 or CaHSF8. **a**, Phylogenetic analysis of the five tested NLRs with its orthologs in *N. benthamiana*. **b**, The distribution of CaKAN3 and CaHSF8 responsive cis-elements and the fragments corresponding to primers used in ChIP-qPCR or ChIP-PCR assay in the promoters of NLR genes in *N. benthamiana*. **c**, The result of ChIP-PCR showed that both CaKAN3 and CaHSF8 bound the promoters of all of the five tested NLR genes. The experiment was carried out once. **d**, The data from ChIP-qPCR showed that both CaKAN3 and CaHSF8 bound the promoters of all of the five tested NLR genes. The enrichment levels of the tested genes were compared with those in the control, and the relative enrichment of IP: Anti-GFP/CaKAN3-GFP or IP: Anti-GFP/CaHSF8-GFP was set to a value of 1 after normalization by input. Data represent the mean ± SD of three biological replicates. Asterisks above the bars indicate significant differences between means ($P < 0.01$) by Fisher's protected t test. **e**, The data from RT-qPCR showed that the overexpression of both CaKAN3 and CaHSF8 significantly upregulated the five tested NLR genes in *N. benthamiana* plants challenged with HTHH at 1 hpt. Data represent the mean ± SD of four biological replicates. The transcript levels of WT were set to 1, *NbActin* was used as an internal control, and different capital letters above the bars indicate significant differences ($P < 0.01$) by Fisher's protected LSD test. In **d**, to **e**, source data are provided as a Source Data file.

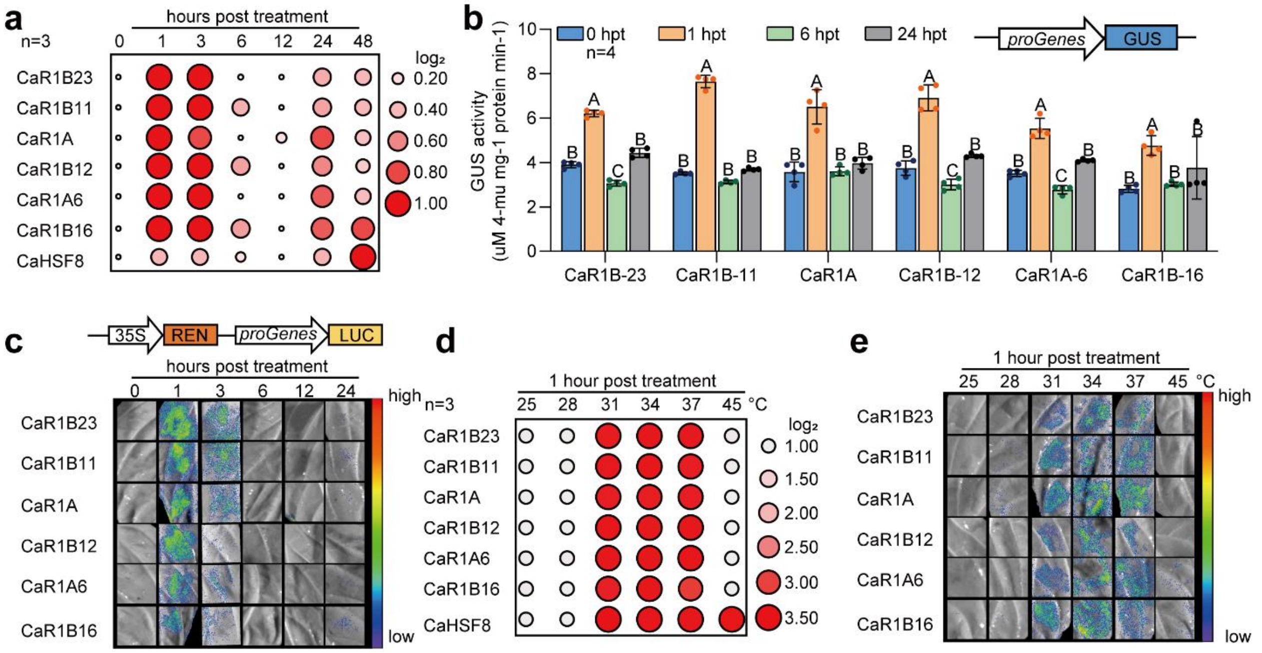

Supplementary Data Fig.15|The transcript levels of *CaR1B23, CaR1B12, CaR1A, CaR1B11, CaR1A6* and *CaR1B16* at different time points after high temperature treatment and treatment with different temperatures. a,*CaR1B23*, *CaR1B12*, *CaR1A*, *CaR1B11*, *CaR1A6*, *CaR1B16* and *CaHSF8* were upregulated at 1 hpt of high temperature treatment. **b**, The transcript levels of *CaR1B23*, *CaR1B12*, *CaR1A*, *CaR1B11*, *CaR1A6* and *CaR1B16* upon high temperature (37 ℃, 90% humidity) treatment at 0, 1, 6, and 24 hpt by GUS activity assay. Data represent the mean ± SD of four biological replicates. Different capital letters above the bars indicate significant differences (P< 0.01) by Fisher's protected LSD test. **c**, The transcript levels of *CaR1B23*, *CaR1B12*, *CaR1A*, *CaR1B11*, *CaR1A6* and *CaR1B16* upon high temperature treatment (37 ℃, 90% humidity) at 0, 1, 3, 6, 12, and 24 hpt by a LUC assay. **d**,*CaR1B23*, *CaR1B12*, *CaR1A*, *CaR1B11*, *CaR1A6*, *CaR1B16* and *CaHSF8* were upregulated by different high temperatures (from 31 to 37 ℃, 90% humidity) treatment at 1 hpt. In **a**, and **d**, means (n = 4) of fold induction compared to 0 hpt treatment shown as log2 were used to construct the heatmap using TBtools. **e**, The transcripti levels of *CaR1B23*, *CaR1B12*, *CaR1A*, *CaR1B11*, *CaR1A6* and *CaR1B16* upon high temperature(25, 28, 31, 34, 37, 45 ℃, 90% humidity) at 1 hpt by LUC assay. In **a**, **b**, and **d**, source data are provided as a Source Data file.

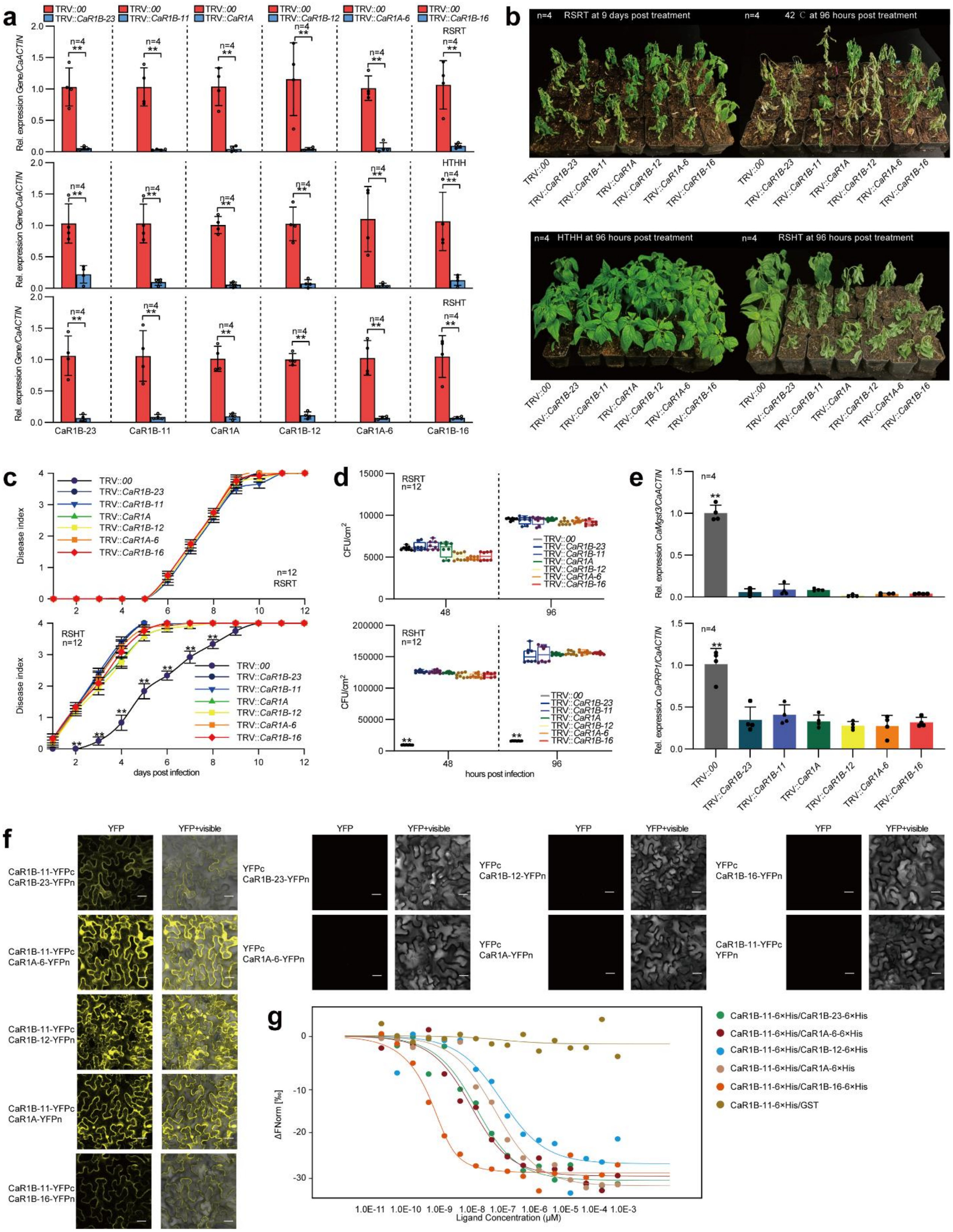

Supplementary Data Fig.16|NLRs acted positively in pepper immunity against RSHT. a, The success of NLR silencing by virus-induced gene silencing (VIGS) by measuring the

transcript levels of NLRs in RSRT-, HTHH- or RSHT-challenged TRV:NLR pepper plants at 1 hour post-treatment (hpt). **b**, Effect of NLR silencing on the response of pepper plants to RSRT and RSHT treatment at 3 and 9 dpt. **c**, The disease indices of *CaKAN3*-silenced pepper plants challenged with RSRT or RSHT from 0 to 12 dpt. The experiment was carried out twice with similar results. Data are shown as the means ± standard errors of 24 replicates. Asterisks above the bars indicate significant differences between means ($P< 0.01$), as calculated with a t test. **d**, The growth of *R. solanacearum* in *R. solanacearum*-inoculated NLR-silenced plants at room temperature or under HTHH, shown as colony-forming units (cfu). Data are shown as the means ± standard errors of eight replicates. Asterisks above the bars indicate significant differences between means ($P< 0.01$), as calculated with a t test. The center line represents the median value and the boundaries indicate the 25th percentile (upper) and the 75th percentile (lower). Whiskers extend to the largest and smallest value. **e**, Relative transcript levels of *CaMgst3* and *CaPRP1* in TRV:*00* and TRV:NLRs pepper plants challenged by RSHT. **f**, The result of BiFC showed that CaR1B-11 interacted with CaR1B23, CaR1A-6, CaR1B12, CaR1A, CaR1B16 in the epidermal cells of *N. benthamiana* leaves by agroinfiltration based transient overexpression at 48 hpi. The experiment was carried out once. **g**, CaR1B-11 interacted with CaR1B23, CaR1A-6, CaR1B12, CaR1A, CaR1B16 by MST, all of the proteins were expressed in and isolated from *E.coli* strain BL21 and the isolated CaR1B-11 was labeled with red fluorescence using a kit provided by manufactory (Monolith NT.115). In **a**, and **e**, The transcript levels of TRV:*00* were set to 1, *CaActin* was used as an internal control, and asterisks above the bars indicate significant differences between means ($P< 0.01$), as calculated with Fisher's protected t test. In **b**, and **c**, the experiments were carried out three with similar results. In **a**, **c**, **d**, and **e**, source data are provided as a Source Data file.

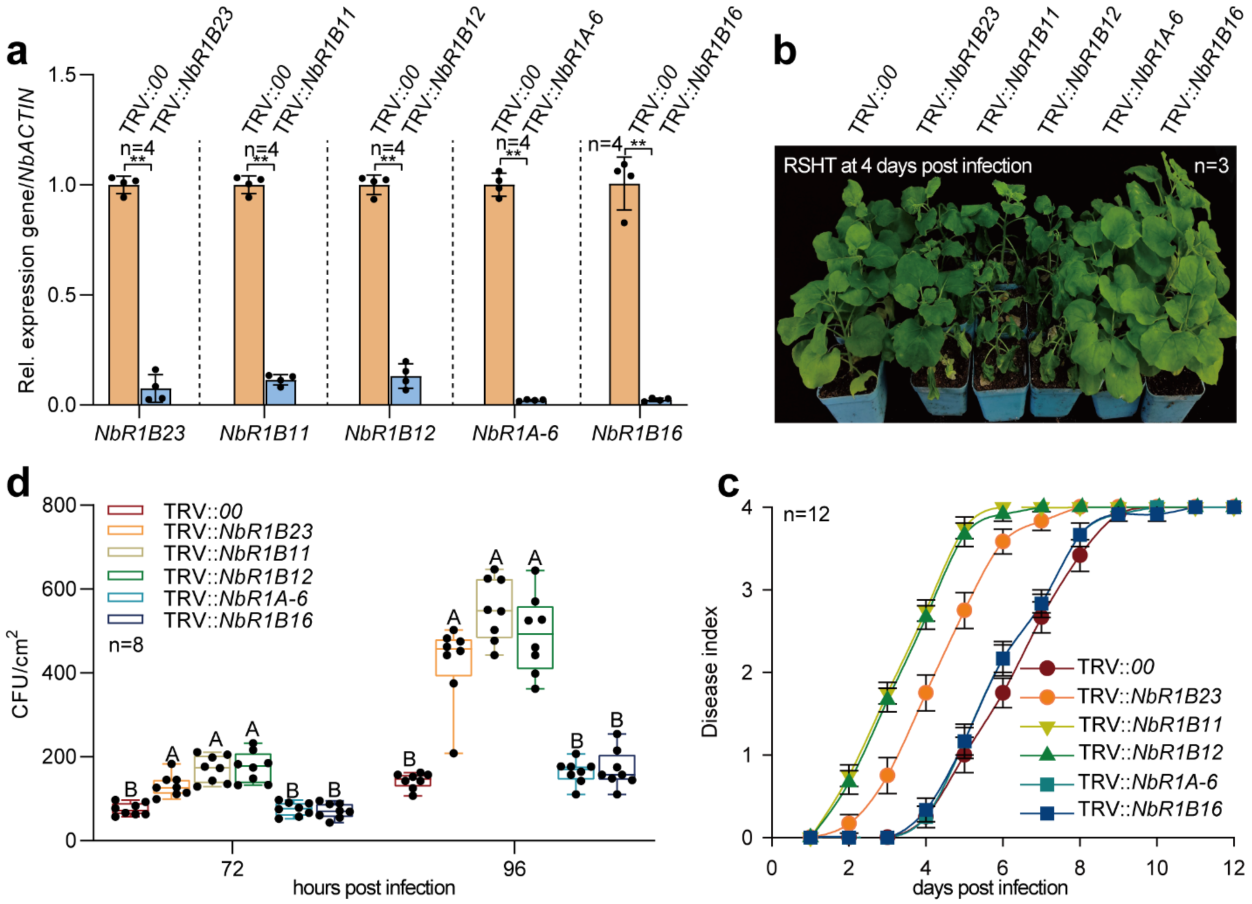

Supplementary Data Fig.17|The effect of NLRs silencing on the response of *N. benthamiana* plant to RSHT. a, The confirmation of NLR gene silencing by RT-qPCR, the result showed the all of the NLR genes were successfully silenced by VIGS. Data represent the mean ± SD of four biological replicates. The transcript levels of TRV::*00* plants were set to 1, *NbActin* was used as an internal control, and asterisks above the bars indicated significant differences between means (*P*< 0.01) by Fisher's protected t test. **b**, The silencing of *NbR1B23*, *NbRIB11* or *NbR1B12* significantly increased *N. benthamiana* susceptibility to RSHT, and the silencing of the other two NLR genes did not produced any phenotypic effect. **c**, The silencing of *NbR1B23*, *NbRIB11* or *NbR1B12* significantly increased the dynamic disease index of *N. benthamiana* plants challenged with RSHT from 0 to 12 dpt, the silencing of other two NLR genes did not affect the dynamic disease index. Data represent the mean ± SD of twelve biological replicates. **d**, The silencing of *NbR1B23*, *NbRIB11* or *NbR1B12* significantly promoted the bacterial growth in leaves of *N. benthamiana* plants challenged with RSHT at 72 and 96 hpt. Different capital letters above the bars indicate significant differences (*P*< 0.01) by Fisher's protected LSD test. The center line represents the median value and the boundaries indicate the 25th percentile (upper) and the 75th percentile (lower). Whiskers extend to the largest and smallest value. In **b**, and **c**, the experiments were carried out twice with similar results. In **a**, **c**, and **d**, source data are provided as a Source Data file.

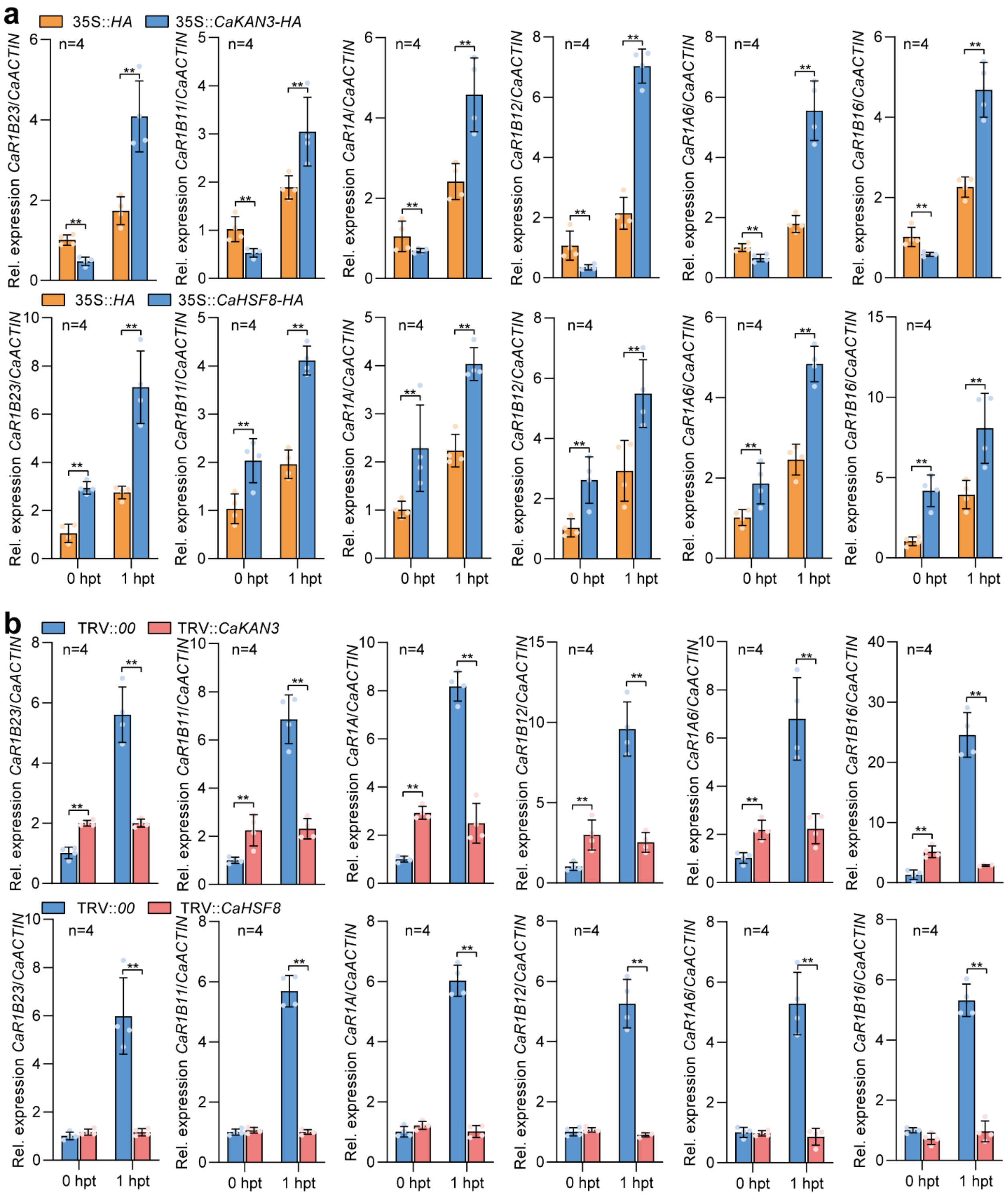

Supplementary Data Fig.18|Regulation of the transcription of the six tested NLR genes by CaKAN3 and CaHSF8. a, The effect of CaKAN3 or CaHSF8 transient overexpression on the transcript levels of the six tested NLR genes in pepper leaves treated with condition of 37 ℃ and 90% humidity at 0 and 1 hpt by RT-qPCR. **b**, Relative transcript levels of NLRs in TRV:*00* and

TRV:*CaKAN3* or TRV:*CaHSF8* pepper plants challenged by condition of 37 ℃ and 90% humidity at 0 and 1 hpt. In **a** and **b**, the transcript levels of TRV:*00*/0 hpt were set to 1,and *CaActin* was used as an internal control. Data are shown as the means ± standard errors of four replicates. Asterisks above the bars indicate significant differences between means ($P < 0.01$), as calculated with a t test. In **a**, and **b**, source data are provided as a Source Data file.

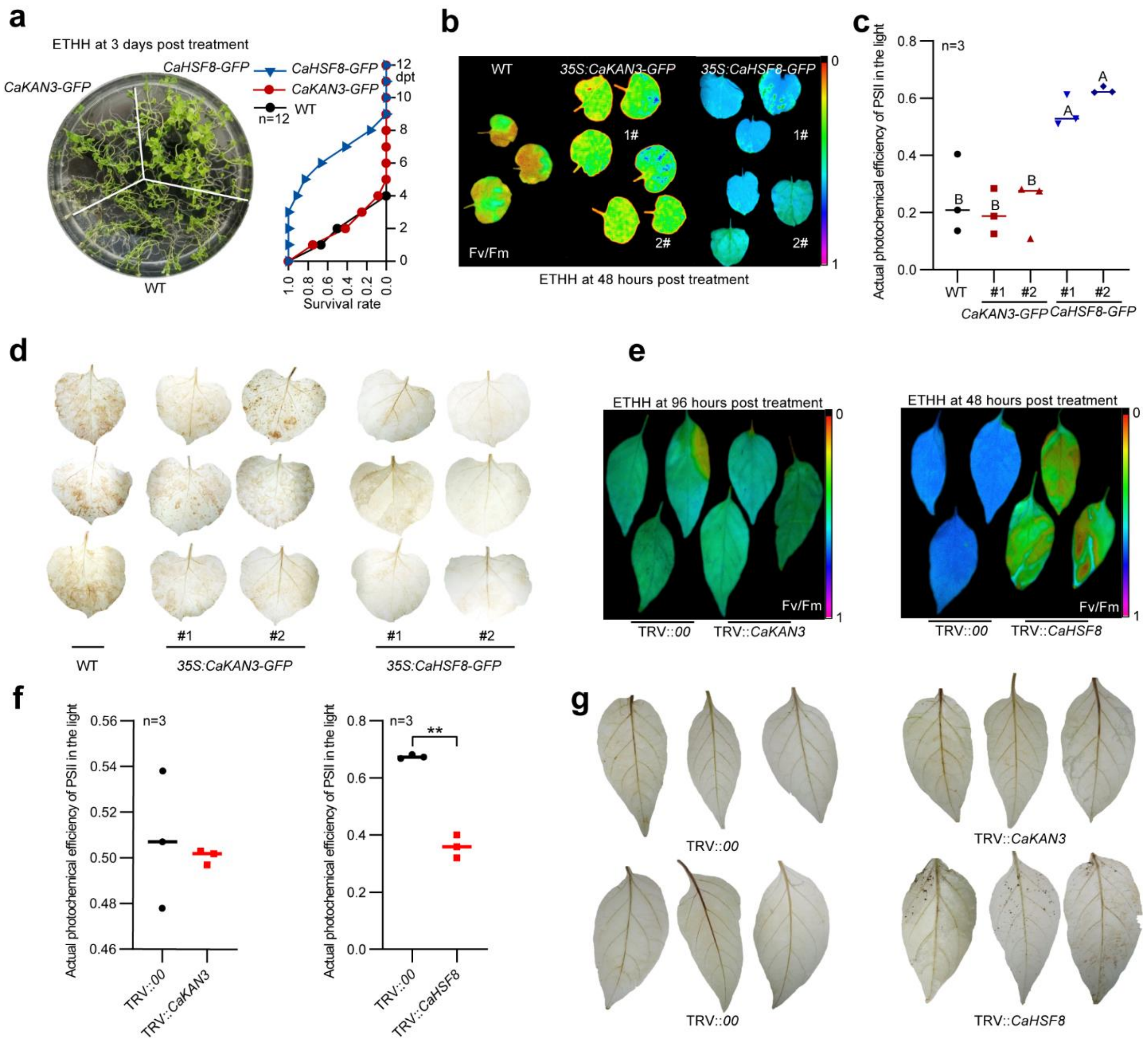

Supplementary Data Fig.19|CaHSF8 acted positively in pepper thermotolerance. **a**, *CaHSF8* overexpressing *N. benthamiana* plants displayed enhanced thermotolerance compared to the wild type control plants. The experiments were carried out twice with similar results. **b**, and **c**, *CaHSF8* overexpressing *N. benthamiana* plants showed higher Fv/Fm or △F/Fm upon ETHH (42 ℃, 90% humidity) treatment. **d**, *CaHSF8* overexpressing *N. benthamiana* plants showed lower level H_2O_2 accumulation displayed by DAB staining upon ETHH (42 ℃, 90% humidity) compared to the wild type control plants. **e**, and **f**, *CaHSF8*-silencing pepper plants showed lower Fv/Fm or △F/Fm upon ETHH (42 ℃,90% humidity) compared to the wild type control plants. **g**, *CaHSF8*-silenced pepper plants accumulated higher level of H_2O_2 accumulation displayed by DAB staining upon ETHH (42 ℃, 90% humidity) treatment. In **a**, **c**, and **f**, source data are provided as a Source Data file.

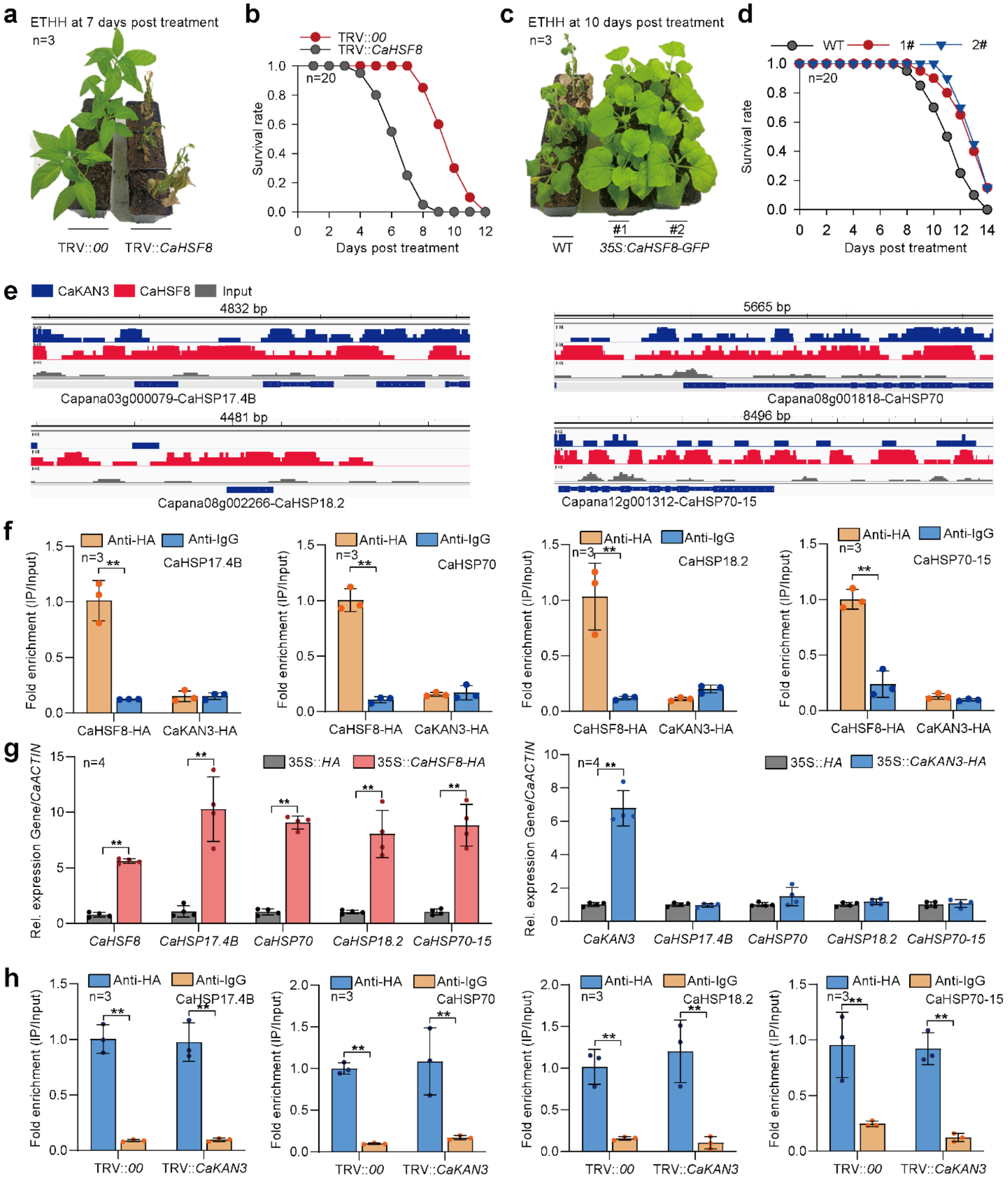

Supplementary Data Fig.20|Direct regulation of a subset of HSP genes by CaHSF8 alone in pepper plants upon ETHH. a, *CaHSF8* silencing pepper plants displayed reduced thermotolerance upon ETHH (42 ℃, 90% humidity). **b**, Mortality of pepper plants upon ETHH from 0 to 12 dpt (days post-treatment). **c**, *CaHSF8* overexpressing *N. benthamiana* plants displayed increased thermotolerance upon ETHH compared to the wild type control plants. **d**,

Mortality of the two *CaHSF8* overexpressing *N. benthamiana* lines, *CaHSF8*#1 and *CaHSF8*#2 upon ETHH, 12 plants of each line were calculated. **e**, Integrative Genomics Viewer (IGV) images of ChIP-seq showed that the promoters of *CaHSP17.4B*, *CaHSP70*, *CaHSP18.2* and *CaHSP70-5* were targeted by CaHSF8. **f**, The data from ChIP-qPCR showed that *CaHSP17.4B*, *CaHSP70*, *CaHSP18.2* and *CaHSP70* were directly targeted by CaHSF8. The relative enrichment of Anti-HA/CaHSF8-HA was set to a value of 1 after normalization by input. Data are shown as the means ± standard errors of three replicates. Asterisks above the bars indicate significant differences between means ($P < 0.01$), as calculated with a t test. **g**,*CaHSP17.4B*, *CaHSP70*, *CaHSP18.2* and *CaHSP70-5* are upregulated by the transient overexpression of *CaHSF8* in pepper leaves. Data are shown as the means ± standard errors of four replicates. Asterisks above the bars indicate significant differences between means ($P < 0.01$), as calculated with a t test. **h**, The silencing of *CaKAN3* did not reduce the targeting of *CaHSP17.4B*, *CaHSP70*, *CaHSP18.2* and *CaHSP70-5* by CaHSF8.The relative enrichment of Anti-HA/TRV::*00* was set to a value of 1 after normalization by input. Data are shown as the means ± standard errors of three replicates. Asterisks above the bars indicate significant differences between means ($P < 0.01$), as calculated with a t test. In **a**, to **d**, the experiments were carried out twice with similar results. In **b**, **d**, **f**, **g**, and **h**, source data are provided as a Source Data file.

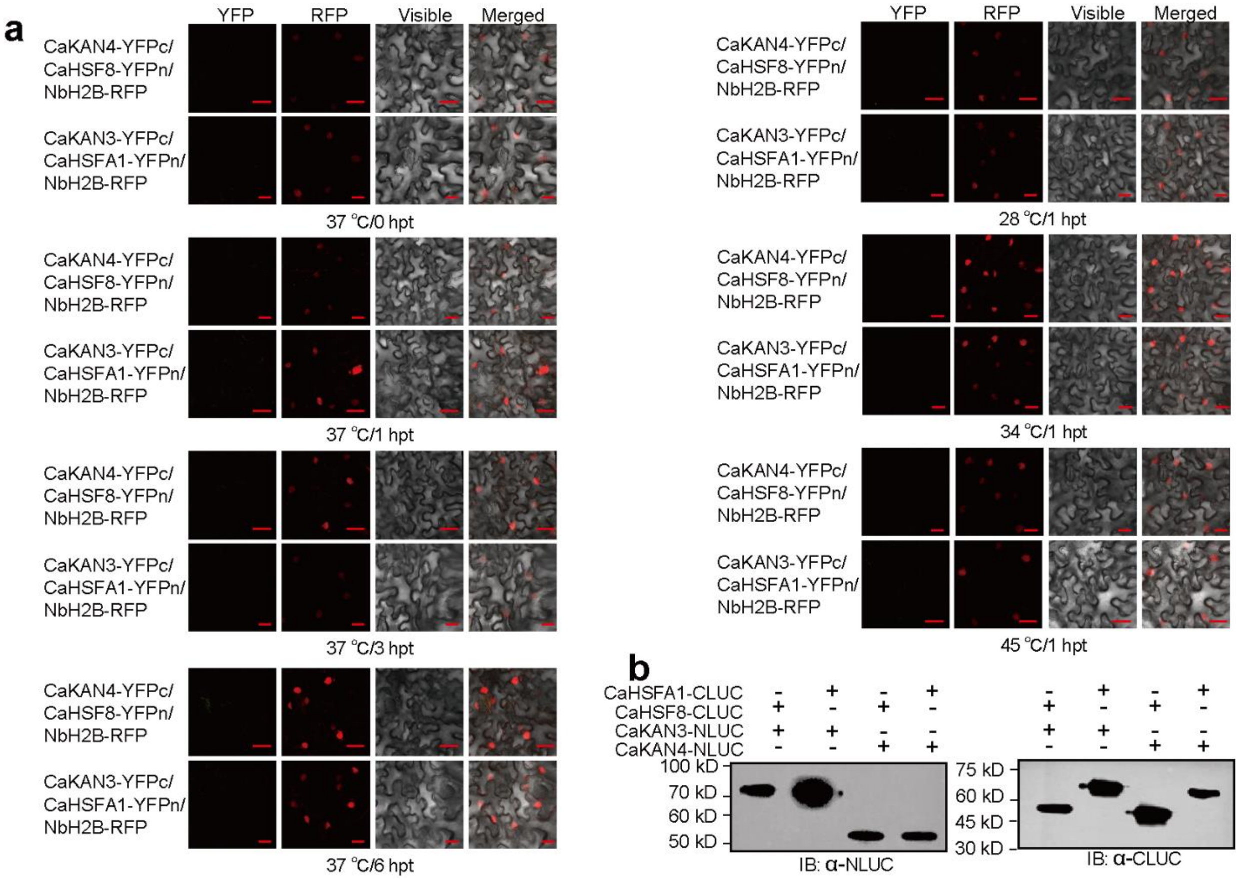

Supplementary Data Fig.21| The negative controls in Fig. 5a and the success confirmation of transient overexpression in Fig. 5b. a, The negative controls in BiFC assay in Fig. 5a. **b**, The success of transient overexpression of CaHSFA1-GLUC,CaHSF8-CLUC, CaKAN3-NLUC and CaKAN4-NLUC in pepper leaves by immunoblotting using antibody of NLUC or CLUC in Fig. 5b. The experiment was carried out once.

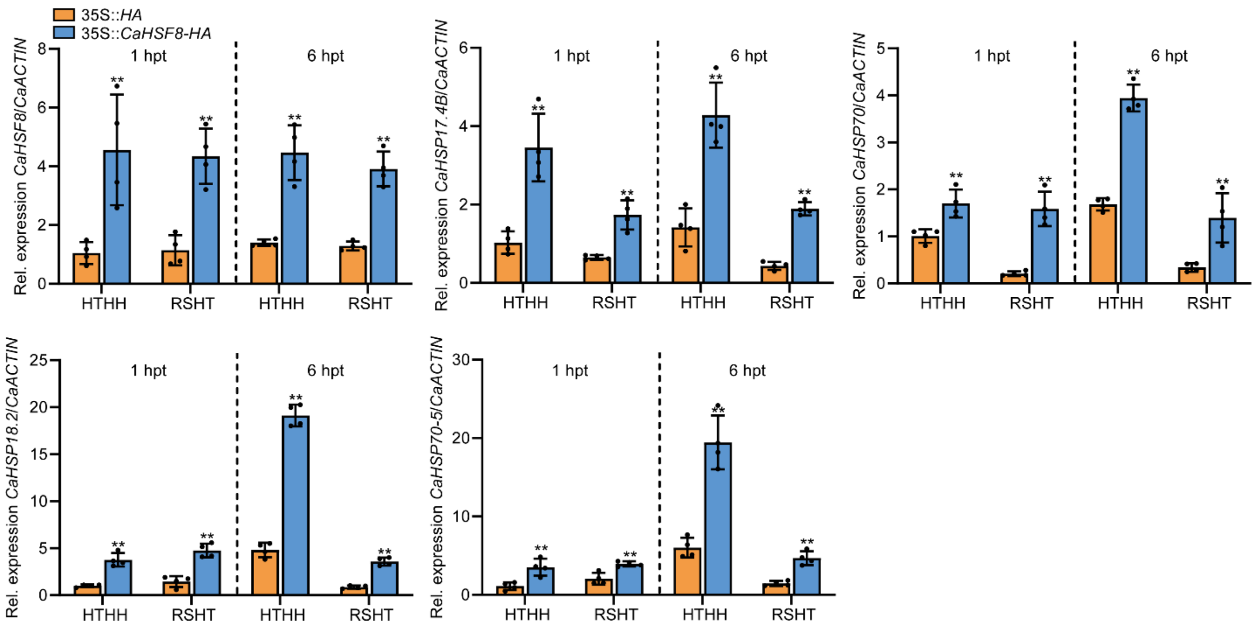

Supplementary Data Fig.22|Transient overexpression of CaHSF8 clearly upregulated the four HSP genes in pepper plants challenged HTHH or RSHT at 1 and 6 hpt. The effect of transient overexpression of CaHSF8 on the transcript levels of HSPs in pepper leaves upon HTHH or RSHT at 1 and 6 hpt by RT-qPCR. The transcript levels of 35S:00 plants upon HTHH at 1 hpt were set to 1,and *CaActin* was used as an internal control. Data are shown as the means ± standard errors of four replicates. Asterisks above the bars indicate significant differences between means (*P*< 0.01), as calculated with a t test. Source data are provided as a Source Data file.

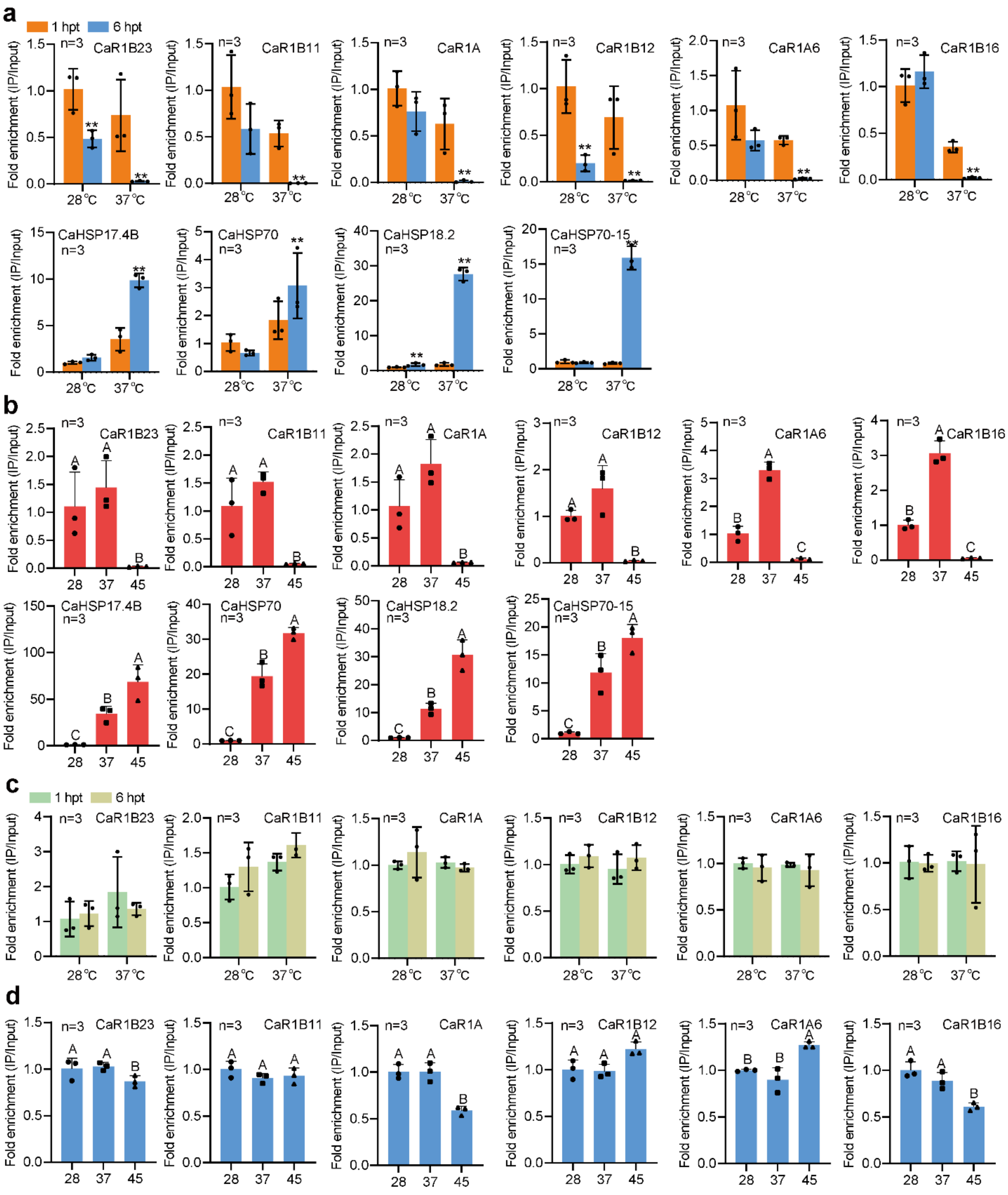

Supplementary Data Fig.23|Enrichment of CaKAN3 and CaHSF8 to the promoters of NLR and HSP genes upon RTHH and HTHH at 1 and 6 hpt or at 1 hpt upon different temperature (28 °C, 37 °C, and 45 °C, 90% humidity). a, The enrichment of CaHSF8 in the promoters of the 6 NLR genes and 4 HSP genes, the chromatins used for ChIP-qPCR were isolated from CaHSF8 transiently overexpressing pepper plants challenged with RTHH (28 °C,

90% humidity) and HTHH (37 ℃, 90% humidity) at 1 and 6 hpt, the relative enrichment in pepper plants upon RTHH at 1 hpt was set to a value of 1 after normalization by input. Asterisks above the bars indicate significant differences between means (P< 0.01), as calculated with a t test. **b**, The enrichment of CaHSF8 in the promoters of the 6 tested NLR genes and 4 tested HSP genes, the chromatins used for ChIP-qPCR were isolated from leaves of pepper plants upon RTHH, HTHH or ETHH (45 ℃, 90% humidity) at 1 hpt, the relative enrichment of CaHSF8 in the promoters upon RTHH at 1 hpt was set to a value of 1 after normalization by input. **c**, The enrichment of CaKAN3 in the promoters of the 6 NLR genes, the chromatins used for ChIP-qPCR were isolated from CaKAN3 transiently overexpressing pepper plants challenged with RTHH or HTHH at 1 and 6 hpt. The relative enrichment of CaKAN3 to the six tested promoters upon RTHH at 1 hpt was set to a value of 1 after normalization by input. **d**, The enrichment of CaKAN3 in the promoters of the 6 tested NLR genes, the chromatins used for ChIP-qPCR were isolated from leaves of pepper plants upon RTHH or HTHH at 1 hpt, the relative enrichment of KAN3 to the 6 tested NLR gene upon RTHH at 1 hpt was set to a value of 1 after normalization by input. In **a** to **d**, data were shown as the means ± standard errors of three replicates; different capital letters above the bars indicate significant differences (P< 0.01) by Fisher's protected LSD test. In **a**, and **b**, the experiments were carried out twice with similar results. In **a**, to **d**, source data are provided as a Source Data file.

References

1 Yang, S. *et al.* Solanaceous plants switch to cytokinin-mediated immunity against Ralstonia solanacearum under high temperature and high humidity. *Plant, cell & environment* **45**, 459-478, doi:10.1111/pce.14222 (2022).

2 Dang, F. *et al.* CaWRKY40, a WRKY protein of pepper, plays an important role in the regulation of tolerance to heat stress and resistance to Ralstonia solanacearum infection. *Plant, cell & environment* **36**, 757-774, doi:10.1111/pce.12011 (2013).

3 Shen, L. *et al.* Pepper CabZIP63 acts as a positive regulator during Ralstonia solanacearum or high temperature-high humidity challenge in a positive feedback loop with CaWRKY40. *Journal of experimental botany* **67**, 2439-2451, doi:10.1093/jxb/erw069 (2016).
