# Supplementary material for: Differential CaKAN3-CaHSF8 associations underlie distinct immune and heat responses under high temperature and high humidity conditions
Source: Nat Commun. 2023 Jul 25;14:4477. doi: 10.1038/s41467-023-40251-8 (PMC10368638; doi:10.1038/s41467-023-40251-8)
Supplement: Supplementary file 3 — Reporting Summary [file 41467_2023_40251_MOESM3_ESM.pdf]

nature portfolio

Corresponding author(s): Prof. Shuilin He

Last updated by author(s): May 27, 2023

Reporting Summary

Nature Portfolio wishes to improve the reproducibility of the work that we publish. This form provides structure for consistency and transparency in reporting. For further information on Nature Portfolio policies, see our Editorial Policies and the Editorial Policy Checklist.

Please do not complete any field with "not applicable" or n/a. Refer to the help text for what text to use if an item is not relevant to your study.
For final submission: please carefully check your responses for accuracy; you will not be able to make changes later.

Statistics

For all statistical analyses, confirm that the following items are present in the figure legend, table legend, main text, or Methods section.

n/a	Confirmed	
☐	☒	The exact sample size (*n*) for each experimental group/condition, given as a discrete number and unit of measurement
☐	☒	A statement on whether measurements were taken from distinct samples or whether the same sample was measured repeatedly
☐	☒	The statistical test(s) used AND whether they are one- or two-sided<br>*Only common tests should be described solely by name; describe more complex techniques in the Methods section.*
☒	☐	A description of all covariates tested
☐	☒	A description of any assumptions or corrections, such as tests of normality and adjustment for multiple comparisons
☐	☒	A full description of the statistical parameters including central tendency (e.g. means) or other basic estimates (e.g. regression coefficient) AND variation (e.g. standard deviation) or associated estimates of uncertainty (e.g. confidence intervals)
☐	☒	For null hypothesis testing, the test statistic (e.g. *F*, *t*, *r*) with confidence intervals, effect sizes, degrees of freedom and *P* value noted<br>*Give P values as exact values whenever suitable.*
☒	☐	For Bayesian analysis, information on the choice of priors and Markov chain Monte Carlo settings
☒	☐	For hierarchical and complex designs, identification of the appropriate level for tests and full reporting of outcomes
☒	☐	Estimates of effect sizes (e.g. Cohen's *d*, Pearson's *r*), indicating how they were calculated

Our web collection on statistics for biologists contains articles on many of the points above.

Software and code

Policy information about availability of computer code

Data collection	Capsicum annuum Zunla Genome version 2.0 (for gene cloned and ChIP-seq analysis), Capsicum annuum protheome from UniProt database (for LcMS/MS).
Data analysis	The data were analyze using GraphPad Prism 9.0, DPS 9.5, Monolith NT.115, TBtools 1.098761, Proteome Discover 2.1, IGV 2.10, Burrows Wheeler Aligner (BWA 0.7.12-r1039), MACS2 2.1.0, MEME 5.4.1 and Tree browser from solgenomics.

For manuscripts utilizing custom algorithms or software that are central to the research but not yet described in published literature, software must be made available to editors and reviewers. We strongly encourage code deposition in a community repository (e.g. GitHub). See the Nature Portfolio guidelines for submitting code & software for further information.

Data

Policy information about availability of data

All manuscripts must include a data availability statement. This statement should provide the following information, where applicable:

- Accession codes, unique identifiers, or web links for publicly available datasets
- A description of any restrictions on data availability
- For clinical datasets or third party data, please ensure that the statement adheres to our policy

The data that support the findings of this study are openly available in [Genome Warehouse in National Genomics Data Center, https://ngdc.cncb.ac.cn/gsa/], under accession number PRJCA018309.

Human research participants

Policy information about studies involving human research participants and Sex and Gender in Research.

Reporting on sex and gender	N/A.
Population characteristics	N/A
Recruitment	N/A
Ethics oversight	N/A

Note that full information on the approval of the study protocol must also be provided in the manuscript.

Field-specific reporting

Please select the one below that is the best fit for your research. If you are not sure, read the appropriate sections before making your selection.

☒ Life sciences ☐ Behavioural & social sciences ☐ Ecological, evolutionary & environmental sciences

For a reference copy of the document with all sections, see nature.com/documents/nr-reporting-summary-flat.pdf

Life sciences study design

All studies must disclose on these points even when the disclosure is negative.

Sample size	In order to ensure the reliability of the experiment, we used different sample sizes for different experiments. In the qPCR and ROS measurements, we collected 4 samples, with one plant in each, all of the plants exhibited similar growth state. In the CFU and Disease index assay, we used 8 and 12 plants, respectively, all the plants exhibited similar growth state, the data were collected and calculated individually. In ChIP-qPCR assay, we used 3 samples, with one plant in each sample, all of the plants used exhibited similar growth state.
Data exclusions	No data was excluded.
Replication	Except for ChIP-seq and Lc-MS/MS, all the other experimental results have undergone at least two independent repeated experiments to ensure reliability.
Randomization	Samples were grown on the same condition and randomly allocated in the growth chamber. Experimental plant materials were collected randomly without any bias.
Blinding	No blinding. The experimental materials are plants, thus the blinding design is not applicable to this system.

Reporting for specific materials, systems and methods

We require information from authors about some types of materials, experimental systems and methods used in many studies. Here, indicate whether each material, system or method listed is relevant to your study. If you are not sure if a list item applies to your research, read the appropriate section before selecting a response.

Materials & experimental systems

n/a	Involved in the study
☐	☒ Antibodies
☒	☐ Eukaryotic cell lines
☒	☐ Palaeontology and archaeology
☒	☐ Animals and other organisms
☒	☐ Clinical data
☒	☐ Dual use research of concern

Methods

n/a	Involved in the study
☐	☒ ChIP-seq
☒	☐ Flow cytometry
☒	☐ MRI-based neuroimaging

Antibodies

Antibodies used	For ChIP assay, 1/1000 GFP antibodies (Abcam,AB290) and 1/1000 IgG antibodies (Emd Millipore, 12370) were used. For WB, 1/5000 Anti-HA tag Antibody- ChIP Grade (Abcam, AB9110) and 1/5000 Tag-His-Tag Antibody (HRP Conjugated) (Abmart, M20020), 1/5000 GST-Tag antibodies (Abmart, M20007), 1/1000 Anti-Myc-Tag Antibody (HRP Conjugated) (Abmart, M20019) , 1/2000 Anti-DYKDDDDK-Tag Antibody (HRP Conjugated) (Abmart, M20008) were used.
Validation	The validation statements for all the commercial antibodies used in this study can be found in the manufacturers' websites through catalogue numbers. These antibodies have been widely and successfully used for ChIP in many studies, for example, Qi H et al.,2020 plant cell; Zhuang H et al.,2020 plant cell; Liu F et al.,2020 plant cell; Zhang B et al.,2020 ;Nature; Huang B et al.,2021 Nat Commun.

ChIP-seq

Data deposition

☒ Confirm that both raw and final processed data have been deposited in a public database such as GEO.

☒ Confirm that you have deposited or provided access to graph files (e.g. BED files) for the called peaks.

Data access links<br>*May remain private before publication.*	All high-throughput sequencing data have been deposited in the China National GeneBank DataBase, CNGBdb (https://db.cngb.org/cnsa/) (accession number: CNP0001155; CNP0003347).
Files in database submission	CaKAN3_clean_2.fq.gz, CaKAN3_clean_1.fq.gz, CaKAN3_2.fq.gz, CaKAN3_1.fq.gz,CaHSF8_2.fq.gz, CaHSF8_1.fq.gz, CaHSF8_clean_2.fq.gz, CaHSF8_clean_1.fq.gz, IP_1.fq.gz, IP_2.fq.gz.
Genome browser session<br>(e.g. UCSC)	https://software.broadinstitute.org/software/igv/

Methodology

Replicates	2 replicates (a pool of DNA from 12 plants per replicate) were performed in this study.
Sequencing depth	The sequencing depth: 10 G; Length of reads: 150-500 bp<br>Sample Raw_reads Raw_bases(G) Clean_reads Clean_bases(G)<br>CaHSF8 26687595 8.01 26412585 7.75<br>CaKAN3 26430836 7.93 26099237 7.64
Antibodies	GFP antibodies (Abcam,AB290) and IgG antibodies (Emd Millipore, 12370).
Peak calling parameters	macs2 callpeak -t IP.uni.dedup.bam -c Input.uni.dedup.bam -g 3.36E+09 -f AUTO -q 0.05 --nomodel --call-summits
Data quality	Reads were eliminated when the average quality score of all the bases was lower than 15 using fastp. Average reliability of bases was more than 95.5%. number of peak (pvalue<0.005) was 36473 (CaHSF8) and 10661 (CaKAN3).
Software	Trimming: fastp 0.19.11; QC: fastqc V0.11.5; mapping: BWA 0.7.12-r1039 peak calling MACS2 2.1.0; KEGG: KOBAS 3.0; Genome browser: IGV 2.10.

This checklist template is licensed under a Creative Commons Attribution 4.0 International License, which permits use, sharing, adaptation, distribution and reproduction in any medium or format, as long as you give appropriate credit to the original author(s) and the source, provide a link to the Creative Commons license, and indicate if changes were made. The images or other third party material in this article are included in the article's Creative Commons license, unless indicated otherwise in a credit line to the material. If material is not included in the article's Creative Commons license and your intended use is not permitted by statutory regulation or exceeds the permitted use, you will need to obtain permission directly from the copyright holder. To view a copy of this license, visit http://creativecommons.org/licenses/by/4.0/
