# Supplementary figures and images for: Differential CaKAN3-CaHSF8 associations underlie distinct immune and heat responses under high temperature and high humidity conditions
Source: Nat Commun. 2023 Jul 25;14:4477. doi: 10.1038/s41467-023-40251-8 (PMC10368638; doi:10.1038/s41467-023-40251-8)

Fig.2e

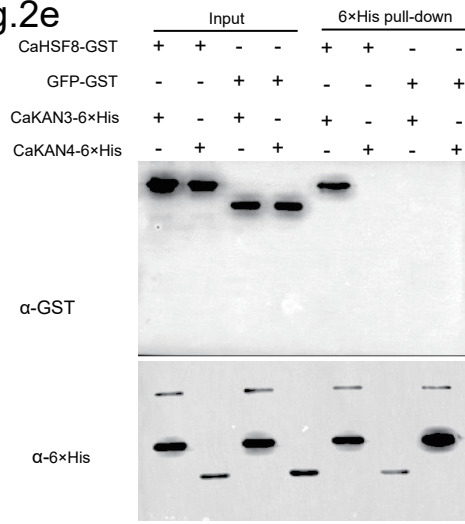

Fig.2f

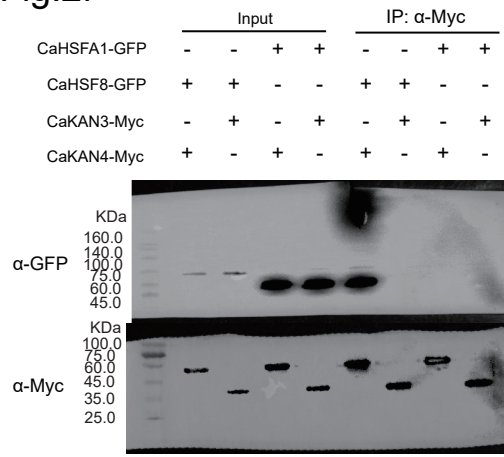

Fig.2g

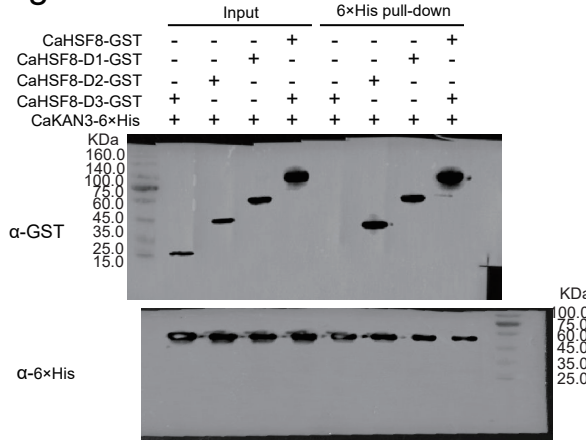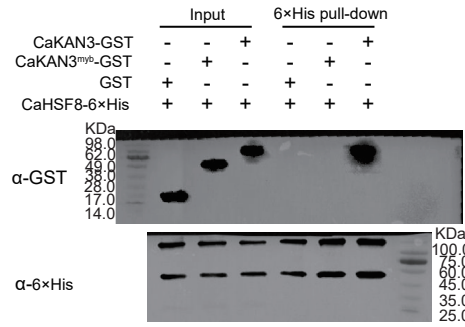

Fig.S4a

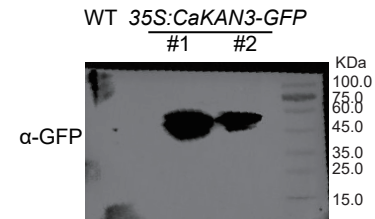

Fig.S5

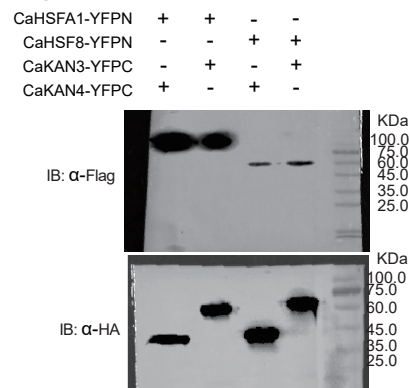

Fig.S8a

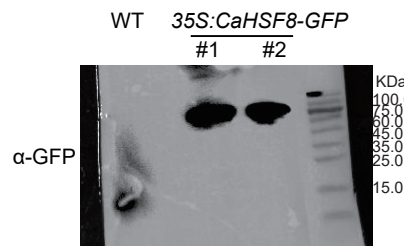

Fig.S9f

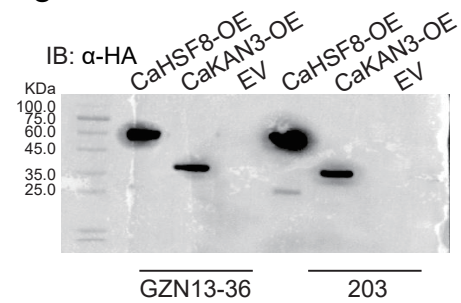

Fig.S21

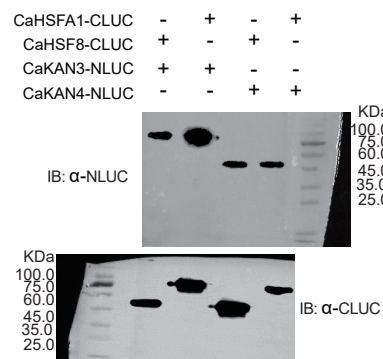

Fig.5d

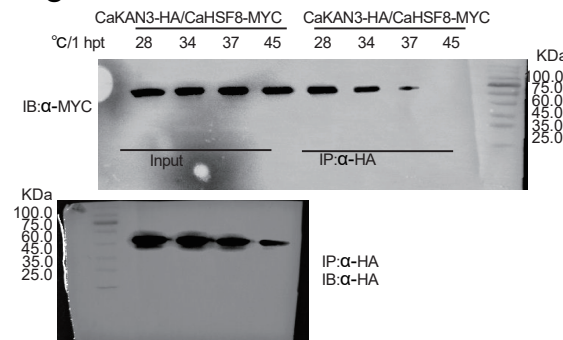

Fig.5e

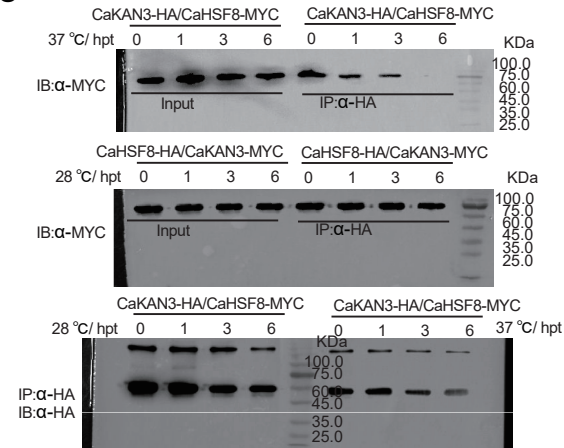

Supplement: Supplementary file 4 — Source Data [file 41467_2023_40251_MOESM4_ESM.zip › Source Data/uncropped immunoblotting images.pdf]
